# Fluorine-free water-in-ionomer electrolytes for sustainable lithium-ion batteries

**DOI:** 10.1038/s41467-018-07331-6

**Published:** 2018-12-14

**Authors:** Xin He, Bo Yan, Xin Zhang, Zigeng Liu, Dominic Bresser, Jun Wang, Rui Wang, Xia Cao, Yixi Su, Hao Jia, Clare P. Grey, Henrich Frielinghaus, Donald G. Truhlar, Martin Winter, Jie Li, Elie Paillard

**Affiliations:** 1 Helmholtz Institute Münster – Forschungszentrum Jülich GmbH (IEK 12), Corrensstrasse 46, 48149 Münster, D-48149 Münster, Germany; 2 State Key Laboratory of Chemical Resource Engineering, Institute of Materia Medica, College of Science, Beijing University of Chemical Technology, Beijing, 100029 P. R. China; 3 Department of Chemistry, University of Cambridge, Lensfield Road, Cambridge, CB2 1EW UK; 4 University Grenoble Alpes, CEA, CNRS, SyMMES, F-38000 Grenoble, France; 5 MEET Battery Research Center, Institute of Physical Chemistry, University of Münster, Corrensstraße 46, D-48149 Münster, Germany; 6 Jülich Centre for Neutron Science at MLZ, Forschungszentrum Jülich GmbH, Lichtenbergstrasse 1, D-85747 Garching, Germany; 7 Department of Chemistry, Chemical Theory Center, and Minnesota Supercomputing Institute, University of Minnesota, Minneapolis, Minnesota 55455-0431 USA; 8 Present Address: Karlsruhe Institute of Technology (KIT), Helmholtzstrasse 11, 89081 Ulm, Germany

## Abstract

The continuously increasing number and size of lithium-based batteries developed for large-scale applications raise serious environmental concerns. Herein, we address the issues related to electrolyte toxicity and safety by proposing a “water-in-ionomer” type of electrolyte which replaces organic solvents by water and expensive and toxic fluorinated lithium salts by a non-fluorinated, inexpensive and non-toxic superabsorbing ionomer, lithium polyacrylate. Interestingly, the electrochemical stability window of this electrolyte is extended greatly, even for high water contents. Particularly, the gel with 50 wt% ionomer exhibits an electrochemical stability window of 2.6 V vs. platinum and a conductivity of 6.5 mS cm^−1^ at 20 °C. Structural investigations suggest that the electrolytes locally self-organize and most likely switch local structures with the change of water content, leading to a 50% gel with good conductivity and elastic properties. A LiTi_2_(PO_4_)_3_/LiMn_2_O_4_ lithium-ion cell incorporating this electrolyte provided an average discharge voltage > 1.5 V and a specific energy of 77 Wh kg^−1^, while for an alternative cell chemistry, i.e., TiO_2_/LiMn_2_O_4_, a further enhanced average output voltage of 2.1 V and an initial specific energy of 124.2 Wh kg^−1^ are achieved.

## Abstract (web summary)

The flammability and toxicity of the currently used electrolytes are the concerns that must be addressed. Here the authors show a non-fluorinated and non-toxic ionomeric aqueous gel electrolyte called water-in-ionomer that allows an enlargement of electrochemical stability window and design of environmentally friendly battery cell chemistries.

## Introduction

Lithium-ion batteries are now used in electric vehicles and are under study for electric grid stabilization to allow for a larger portion of the electric power supply to be derived from renewable, but intermittent, energy sources^1^. However, as battery size increases, so do their environmental impact and associated risks. Besides the toxic and costly transition metals, such as Ni and Co used in cathodes, key concerns are the flammability and toxicity of the electrolyte^2^. Thus, the use of non-flammable and nontoxic electrolytes would be desirable. In recent research, various alternative electrolytes were proposed. In particular, highly concentrated electrolytes having no “free” solvent molecules present characteristics that differ significantly from their “diluted” 1 M counterparts, especially concerning their electrochemical stability window (ESW)^3^. Among them, “polymer-in-salt” electrolytes^4^ were proposed to take advantage of the high solubility of low lattice energy Li salts, such as lithium bis(trifluoromethanesulfonyl)imide (LiTFSI) in polyethylene oxide ^5,6^. Unfortunately, despite some attempts at developing non-fluorinated anions^7–12^, low lattice energy organic Li salts are usually heavily fluorinated, toxic (LiTFSI has a LD50 (oral, rat) of 160 mg kg^−1^, according to the material saftey datasheet of Solvay (https://www.solvay.us/en/binaries/PRC90029263-USA-340548.pdf)), and environmentally persistent. More recently, a variety of solvents, including glymes ^13,14^, cyclic ethers^15^, and acetonitrile^16^, have been used in “solvent-in-salt” electrolytes with LiTFSI as lithium salt. In most cases though, this approach increases the fluorine content of the electrolyte, and although LiTFSI could potentially be recycled^17^, increases the price and toxicity of the electrolyte. Another approach consists in developing a lithium-ion chemistry that would accommodate an aqueous electrolyte^18,19^, which could—in addition to the advantages it brings in terms of safety—overcome the use of expensive and fluorinated anions due to the excellent solvating properties of water. A significant problem, however, is that water limits the ESW. Nevertheless, the 1.23 V “thermodynamic” ESW of water can be exceeded in many cases. For instance, Suo et al.^20^ and Dong et al.^21^ proposed a “water-in-salt” electrolyte with a 21 m solution of LiTFSI in water, later extended to mixtures of perfluorinated Li salts^20,22,23^, providing an outstanding ESW and battery output voltages of 2 to 3 V. Nonetheless, although the flammability issue is solved and the performance greatly improved, the fluorine content is, in those cases, much higher than in conventional lithium-ion electrolytes.

Here, we propose a type of electrolyte: A “water-in-ionomer”, non-fluorinated, and non-toxic ionomeric aqueous gel electrolyte that, although being derived from a weak acid and incorporating a relatively high water fraction, exhibits properties similar to those of “water-in-salt” electrolytes for operating Li-ion batteries with voltages far beyond water ESW.

## Results

### From dry ionomers and “solvent-in-salt” to “water-in-ionomer” electrolytes

Ionomers^24–27^, (i.e., lithium salts with the anionic moiety bound to a polymer backbone), providing that they can offer sufficient Li^+^ mobility, would offer several advantages, such as high Li^+^ transference numbers, and thus limited concentration gradients and Li dendrites growth^28^. One of the greatest challenges for these ionomers, though, is their complex preparation, given that the ionic function should allow for facile dissociation (thus, preferentially incorporating an fluorinated anionic moiety) and, for “dry” polymer electrolytes, one requires interspacing solvating units that simultaneously possesses high segmental mobility to ensure ionic dissociation and conduction. However, when ionomers are mixed with a low-viscosity solvent allowing high dissociation of the ionic moiety and high mobility, there is no longer a need for intrinsic solvation and mobility. Hence, the use of water as plasticizer and co-solvent for ionomers should allow using cheaper and non-fluorinated anionic moieties. This points to single block ionomers, such as polyacrylic acid (PAA) which is inexpensive and commercially widespread (used in disposable diapers) and whose non-toxic sodium salt has been listed as food additive by the FDA^29^.

**Fig. 1:**
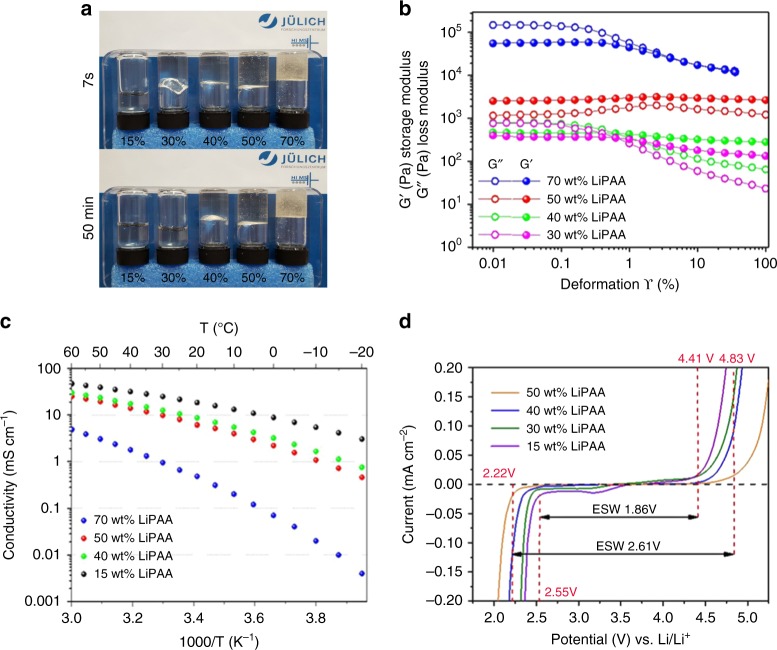
Mechanical and electrochemical properties of the electrolytes. **a** Appearance of LiPAA aqueous electrolytes 7 s and 50 min after flipping the flasks. **b** Storage and loss moduli of the gel electrolytes (from 30 wt% to 70 wt% LiPAA). **c** Arrhenius plot of the conductivities of the electrolytes. **d** Electrochemical stability window with Pt as the working electrode, scan rate: 0.1 mV s^−1^

The lithiated form (LiPAA) PAA was evaluated in aqueous gels. Figure 1a shows that gels with excellent dimensional stability are obtained for 50 wt% of LiPAA and above. However, the 70% gel is rather rigid (which leads to contact issues in cells) and includes bubbles, thus is difficult to process. The evolution of the storage and loss modulus of the gels (Fig. 1b), detailed in Supplementary Note 1, shows that the 50 wt% gel deviates from the general trend, with a more elastic behavior (i.e., tan δ = G’/G” > 1) on the whole deformation range as well as an increase of the storage modulus with increasing deformation. Figure 1c shows the conductivity of the electrolytes which follow a Vogel–Tammann–Fulcher (VTF) behavior (the VTF parameters are reported in Supplementary Table 2 and are discussed in Supplementary Note 2). The 50 wt% LiPAA gel exhibits 6.5 mS cm^−1^ at 20 °C, similarly to organic carbonate-based lithium-ion electrolytes (σ = 5–11 mS cm^−1^)^30^, which is especially high considering that the anionic movement is limited in the electrolyte (a Li^+^ transference number (T^+^) of 0.77 has been determined by pulse-field gradient NMR (PFG-NMR) (see Supplementary Note 3 and Supplementary Table 5 for other T^+^ values). As presented in Fig. 1d, the ESW of the electrolytes evolves with the polymer content in a similar trend as reported for “water-in-salt” electrolytes^20^. A first reduction starts at rather high potential (ca. 3.5 V vs. Li/Li^+^ for the^14^ wt% LiPAA electrolyte). This is above hydrogen evolution and likely related to −COOH reduction^31^, more pronounced in the more diluted electrolytes where water self-ionization (and thus −COOH formation) is more marked. The current and reduction potential both decrease with LiPAA content, either due to PAA/Li/H_2_O interactions, impaired PAA mobility or electrode passivation. Concerning the main oxidation and reductions reactions, a priori linked to water, the trend is rather obvious, with an increase of 300 mV in the cathodic direction and of 400 mV toward oxidation from^14^ wt% to 50 wt% LiPAA. Especially in the anodic direction, a steep increase is observed for the 50 wt% gel which, composition-wise, stands at the border of “water-in-salt*”* and “salt-in-water*”* electrolytes^15^, suggesting a change in water/Li^+−^COO^−^ interactions and self-organization beyond this ratio.

### Water interactions and structural evolutions

Water is both a donor and an acceptor solvent, because it interacts with the Li^+^ cation via its oxygen and with the carboxylate group via its hydrogens. To understand the effect of solvation on the electrochemical stability and local structure, quantum mechanical electronic structure calculations were combined with solid-state nuclear magnetic resonance (ssNMR) and X-ray and neutron scattering characterization (SAXS and WANS). Electrolytes from 84% LiPAA to 10% LiPAA were examined; and the number of H_2_O and D_2_O molecules per carboxylate for intermediate weight percentages is given in Supplementary Table 1.

For modeling, we included a monomer of LiPAA (CH_3_CH_2_COOLi) and up to eight water molecules.

The details of the quantum mechanical simulations, optimized structures of LiPAA(H_2_O)_n_ (*n* = 1–8) clusters, selected bond lengths, energies and free energies, as well as a more detailed analysis are given in Supplementary Note 4 and Supplementary Table 4).

**Fig. 2:**
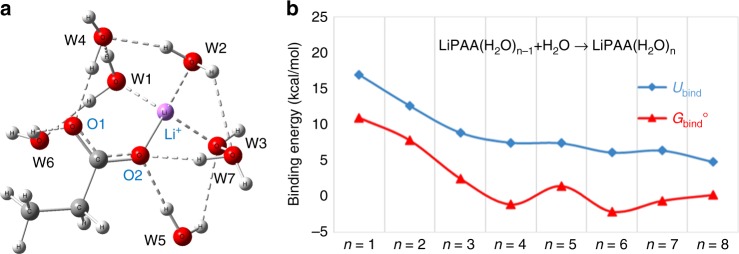
Evolution of LiPAA solvation sphere. **a** Binding order of water molecules with LiPAA as calculated by M08-HX/MG3S/SMD. **b** Binding energies (*U*_bind_) and standard-state binding free energies (*G*_bind_°) of LiPAA(H_2_O)_n_ (*n* = 1–8) as calculated by M08-HX/MG3S/SMD

Figure 2a shows the order in which water molecules would optimally add to a LiPAA monomer. The first two water molecules bind to Li^+^ only, the third binds to Li^+^ and carboxylate oxygens. From the fourth water molecules, H_2_O binds to −PAA and above six water molecules, they bind to carboxylate O and other water molecules. Finally, above eight water molecules, free water is present in all isomers.

Figure 2b and Supplementary Table 4 show the binding energies and binding free energies of LiPAA(H_2_O)_*n*_ (*n* = 1–8). For LiPAA(H_2_O)_*n*_ (*n* = 1–4), the binding energy is gradually reduced as the number of water molecules increases. The binding free energy of the fourth through eighth water may be endergonic because of entropy effects and the gradual saturation of LiPAA.

To understand the role of electrostatics in the water binding process, charge model 5 (CM5)^32^ was used to determine the partial atomic charges on the Li^+^ and the oxygen atoms in the clusters. The partial charge of the lithium ion decreases from 0.7 to 0.5 with increasing coordination of water in LiPAA(H_2_O)_*n*_ (*n* = 1–3). For LiPAA(H_2_O)_*n*_ (*n* = 4–8), the negative charge of carboxylate oxygen atoms decreases with formation of hydrogen bonds between the COO^−^ group and water molecules. This is consistent with the observed increase of anodic stability up to 50 wt% polymer as the water molecules, in this case, interact more with the oxygen atoms, which in turns results in an incremental increase in cathodic stability.

Electrode passivation (i.e., the blocking of the active adsorption site for H_2_ evolution) by either adsorption of polymer or accumulation of reduction products, however, might also play a role in the ESW extension.

**Fig. 3:**
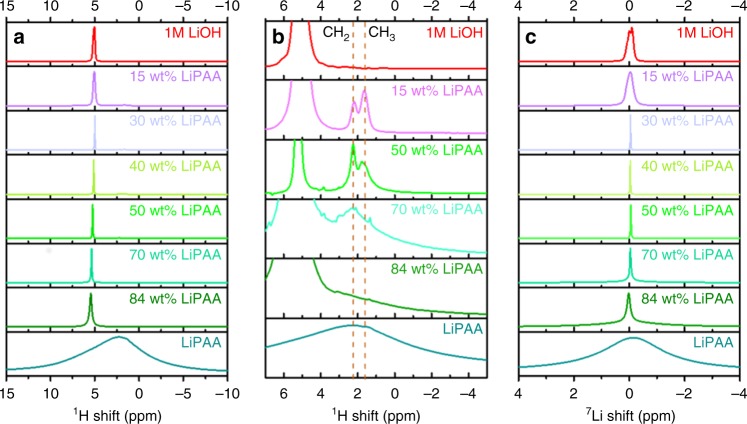
MAS NMR spectra. **a**, **b** ^1^H MAS NMR spectra of LiPAA/H_2_O electrolytes, the dashed lines indicating the shift of the “water” resonance to lower frequency (blue) and the sharpening of the CH_2_ resonance (red) as the water content increases. **c** ^7^Li MAS NMR spectra of the LiPAA/H_2_O electrolytes. All the LiPAA electrolyte spectra were acquired with a spinning rate of 14 kHz while the spectra of 1 M LiOH solution was measured under static condition

The interactions between water and LiPAA were probed via ^1^H NMR and ^7^Li NMR. LiPAA gives rise to a broad ^1^H NMR (Fig. 3a) peak between 12 and −8 ppm that arises from the CH_2_ and CH_3_ groups in LiPAA, the restricted motion and consequently large ^1^H dipole–dipole interactions, and the non-crystalline nature of the LiPAA (which will give rise to a distribution in chemical shifts) resulting in line broadening. The ^7^Li (Fig. 3c) resonance of LiPAA is also broad indicating restricted Li^+^ mobility. The addition of water results in a dramatic reduction in linewidths. As the amount of water in LiPAA increases, the resonances of CH_2_ (2.2 ppm) and CH_3_ (1.6 ppm) start to become better resolved, as marked in Fig. 3b, indicating that the water presumably decreases the packing density of the LiPAA and thus the CH_2_ and CH_3_ groups become more mobile. The shift of ^1^H water in the 84 wt% sample is 0.6 ppm larger than that of free water (4.8 ppm)^33^, suggesting that the water in the sample is bonded to COO^−^ groups and the Li^+^ cations. When the LiPAA content decreases from 84 wt% to 30 wt%, the viscosity of the electrolyte is reduced and the water ^1^H resonance shifts to lower frequency and sharpens, presumably as the hydrogen bonding to the LiPAA COO^−^ groups decrease and the water intermolecular hydrogen bonding increases. The ^7^Li resonance similarly sharpens as the mobility of the Li^+^ ions increases. By 30 wt%, both the ^1^H “water” resonance and the ^7^Li resonance are similar to those of the bulk 1 M LiOH resonances, indicating that free water and Li^+^ ions are present. The peak widths of the 15 wt% sample and 1 M aqueous LiOH ^1^H and ^7^Li resonances are broader than that of the 30 wt% electrolyte, due to problems of shimming these samples in the wide bore magnet. Solution NMR experiments (Supplementary Fig. 1) with improved shimming showed that the linewidth of the ^1^H (water) and ^7^Li resonances were an order of magnitude narrower than those acquired on the ssNMR spectrometer (Supplementary Table 3), the resonances continuing to sharpen slightly from the 15 to 0 wt% LiPAA samples. The ^17^O NMR spectra of the 50 wt% sample also show a broad resonance at 0.3 ppm (Supplementary Fig. 2), similar to the previous results reported for “water-in-salt” electrolyte^20^, suggesting that bound water is present in this 50 wt% sample.

**Fig. 4:**
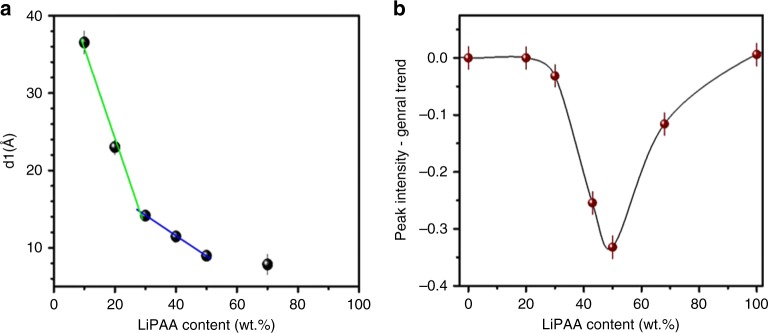
Structural evolution of the electrolytes. **a** SAXS: Evolution of the correlation distances *d*_1_ as a function of the LiPAA content in the electrolytes. **b** WANS**:** Deviation to the trend of the peak intensity for *q*_2_ (at 1.27 Å^−1^, *d*_2_ = 5 Å), displayed as a function of the LiPAA content in D_2_O electrolyte

Small angle X-ray scattering (SAXS) results shown in Supplementary Fig. 3, show one characteristic peak q_1_, corresponding to a distance d_1_ which decreases with the polymer content (Fig. 4a). Thus it can be linked to the local structure, namely, the presence of hydrophilic domains, separated by the polymer chains. However, no second order peak can be seen, due to the lack of long-range order. Interestingly, the evolution of the distance d_1_ with the LiPAA content seems to follow different slopes as the LiPAA content increases, which suggests that different local structural arrangements may correspond to each fraction of the curve, although the lack of second order signal does not allow concluding concerning their exact nature.

Wide angle neutron scattering (WANS) (Supplementary Fig. 4) measurements on LiPAA/D_2_O samples reveal two other characteristic distances: A sharp peak *q*_2_, at 1.27 Å^−1^ (*d*_2_ = 5 Å) and a broader peak *q*_3_ at 2.1 Å^−1^ (*d*_3 = _3.0 Å). *d*_2_ corresponds to the distance between two consecutive carboxylate function facing the same direction on a straight polymer chain. It is not seen in the pure polymer, indicating that the polymer chains straighten to accommodate water. Figure 4b shows the deviation to the general trend for the peak intensity. It can be seen that this linear LiPAA arrangement is far less marked for the 50 wt% sample (3.9 D_2_O per Li^+^). The 50 wt% sample peculiar mechanical properties (elastic behavior on the whole range of deformation), as compared with the other samples, and well-preserved conductivity (vs. 40 wt% LiPAA) could possibly be due to an intermediate solvate structure (i.e., short range structuration) that would form around 50 wt%, where polymer chains are less straight than at lower and higher contents.

The intensity of the broad D_2_O peak *q*_3_ at 2.1 Å^−1^ (*d*_3 = _3.0 Å) is displayed as a function of the water content in Supplementary Fig. 5. Most of the data points follow a linear behavior that correlates with the linear decrease of hydrogen atoms in the sample. *d*_3_, related to oxygen–oxygen correlation, evolves with water solvation and reaches a maximum of deviation to the trend for the 30% LiPAA sample, indicating a maximum distortion of D_2_O organization for this water content. (i.e., for 9.1 D_2_O per Li^+^). This would be due to its interaction with –COO–Li, while at higher LiPAA content, the intensity goes back to the linear trend. This maximum distortion of the water ordering is usually achieved by hydrogen bonding and lithium ion solvation. Below 70 wt% water content, the water is strongly coordinated to the polymer at the oxygen atoms and the lithium ion; at 70 wt% water content, the transition to free water molecules takes place and the structure is distorted most, while above 70 wt% water content, hydrogen bonding is facilitated again due to excess water.

### Sustainable and nontoxic aqueous lithium-ion battery cells

**Fig. 5:**
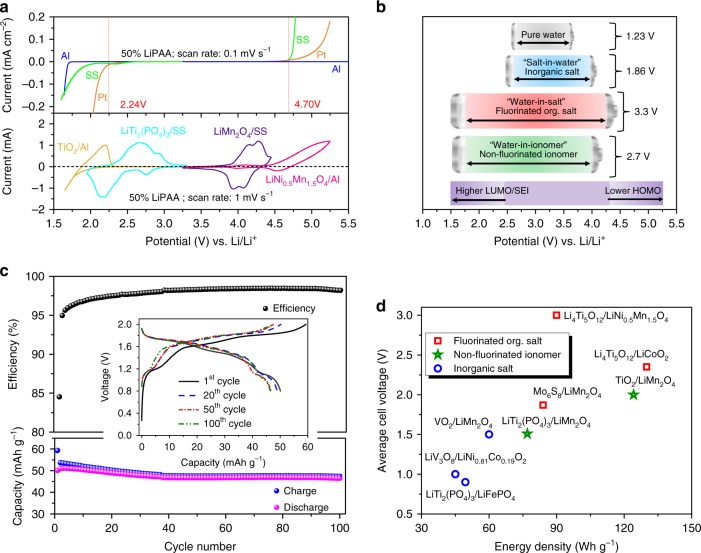
50wt% ‘water-in-ionomer’ gel as lithium-ion electrolyte. **a** Electrochemical stability windows of the 50 wt% LiPAA electrolyte measured on Pt, stainless steel (SS) and Al as well as cyclic voltammograms of TiO_2_, LiTi_2_(PO_4_)_3_, LiMn_2_O_4_ and LiNi_0.5_Mn_1.5_O_4_ on various current collectors as indicated in the graph. Scan rate: 0.1 mV s^−1^. **b** Comparison of aqueous lithium-ion battery end-of-charge voltages with various salts. The “salt-in-water” electrolyte correspond to 1 M Li_2_SO_4_ in a LiTi_2_(PO_4_)_3_/LiMn_2_O_4_ cell^39^, the “water-in-salt electrolyte” corresponds to Li(TFSI)_0.7_(BETI)_0.3_·2H_2_O in a Li_4_Ti_5_O_12_/LiNi_0.5_Mn_1.5_O_4_ cell^22^, and the “water-in-ionomer” corresponds to the 50 wt% LiPAA gel electrolyte in a TiO_2_/LiMn_2_O_4_ cell. **c**. Evolution of specific capacities and coulombic efficiencies of a LiTi_2_(PO_4_)_3_/LiMn_2_O_4_ battery cell at 0.5 C. Current collectors: SS. The weight refers to both electrodes. Insert: Voltage profiles for selected cycles as indicated on the graph. **d** Performance data of aqueous lithium-ion batteries based on various electrochemical couples. LiTi_2_(PO_4_)_3_/LiFePO_4_^40^, LiV_3_O_8_/LiNi_0.81_Co_0.19_O_2_^41^, VO_2_/LiMn_2_O_4_^18^, Mo_6_S_8_/LiMn_2_O_4_^20^, Li_4_Ti_5_O_12_/LiCoO_2_ and Li_4_Ti_5_O_12_/LiNi_0.5_Mn_1.5_O_4_^22^. Insert: Energy densities for selected aqueous electrolytes for LiTi_2_(PO_4_)_3_/ LiMn_2_O_4_ couple. 50 wt% LiPAA, 5M LiNO_3_^42^, 2 M Li_2_SO_4_^43^, 1 M Li_2_SO_4_^45^

Figure 5a shows the variation of the ESW of the 50 wt% LiPAA electrolyte using Pt, stainless steel (SS), and Al electrodes. As can be seen, Al, a typical current collector for Li-ion batteries, allows for an extended ESW as compared with Pt or stainless steel (SS). The electrolyte allows reversible insertion and deinsertion of lithium for both TiO_2_ and LiTi_2_(PO_4_)_3_ anodes. For both electrodes, lithium electro(de)insertion occurs at the same potentials as in conventional organic electrolytes, contrary to TiO_2_ in more concentrated “water-in-salt” electrolytes^23^, which is advantageous in regard to energy density, but more challenging for the electrolyte. LiMn_2_O_4_ delithiation is reversible, but for LiNi_0.5_Mn_1.5_O_4_, only partial delithiation occurs on the reverse scan, probably due to extended self-discharge or other parasitic reactions such as Al anodic dissolution^34^ in the presence of LiNi_0.5_Mn_1.5_O_4_ in aqueous media. In fact, a prior study used Ti as current collector^16^.

Several lithium-ion cell chemistries were assembled using the 50 wt% LiPAA electrolyte. The electrochemical performance of a TiO_2_/LiMn_2_O_4_ cell is described in Supplementary Fig. 6. It delivered 59.2 mAh g^−1^ in the first cycle (with an average output voltage of 2.1 V, corresponding to an energy density of 124.2 Wh kg^−1^). The capacity of the cell decays rapidly, probably due to ineffective (slow) electrode passivation, before stabilizing. In fact, TiO_2_ is known to catalyze the decomposition of water^35^ and usually requires carbon coating for preventing direct contact with H_2_O^36^ and the active sites at its surface. However, the result compares favorably to previous results for non-carbon-coated TiO_2_ using a “water-in-salt” (LiTFSI-based) electrolyte^23^, both in terms of initial delivered capacity and cycling stability. While improvements are still necessary, the coulombic efficiency progressively increases to ca. 98%, which indicates that electrode passivation occurs over cycling.

The performance of a TiO_2_/LiNi_0.5_Mn_1.5_O_4_ cell, cycled between 1.4 V to 3.2 V is shown in Supplementary Fig. 7. It delivered only a small fraction of its theoretical capacity (ca. 23 mAh g^−1^ for the full cell) and, despite an increase of coulombic efficiency with cycling, it only stabilizes to ca. 80%, confirming the voltammetry results for LiNi_0.5_Mn_1.5_O_4_. Given that some water in the electrolyte does not interact with Li^+^, but rather with COO^-^, which has a priori a limited effect on the anodic stability, it seems that the practical gain in anodic stability are limited for the 50 wt% LiPAA electrolyte, even though some cycling at 3.2 V is possible.

## Discussion

Figure 5b compares the thermodynamic ESW of water with the end-of-charge voltages reached with different aqueous Li-ion battery chemistries and either conventional “salt-in-water” aqueous electrolyte with inorganic salts or “water-in-salt” electrolytes with fluorinated salts and the LiPAA 50 wt% electrolyte (“water-in-ionomer” electrolyte) in a TiO_2_/LiMn_2_O_4_ cell. For the latter, the enlargement of the ESW window is mainly due to gains in cathodic stability, which is the main challenge for aqueous electrolytes. On the other hand, fluorinated “water-in-salt” electrolytes allow better anodic stability due to the stronger interactions of water, present in lower amounts, with the Li^+^ ions.

The best cycling stability, shown in Fig. 5c was reached for a LiTi_2_(PO_4_)_3_/LiMn_2_O_4_ cell, which delivered rather stable capacities for 100 cycles with an initial energy density of 77 Wh kg^−1^ and a remarkable capacity retention over 100 cycles. The voltage profiles are hardly affected by the cycling, and efficiencies are close to 100% after stabilization ( > 98.0% from cycle 35 and up to 98.5%). The same cell chemistry cycled using a 40 wt% LiPAA electrolyte is shown in Supplementary Figs. 8–9. The capacity decay is far more marked with only a 77% capacity retention within 100 cycles. Figure 5d compares different aqueous battery chemistries in terms of energy density and average output voltages. The energy density (77 Wh kg^−1^) reached with LiTi_2_(PO_4_)_3_/LiMn_2_O_4_ cells is higher than those generally achieved with similar electrode chemistries in conventional aqueous electrolytes due to a favorable electrode weight ratio and a high-discharge voltage allowed by the high rate capability of the electrolyte and the LiTi_2_(PO_4_)_3_ electrode. On the other hand, the TiO_2_/LiMn_2_O_4_ battery compares well with those obtained with concentrated fluorinated anions.

In summary, the series of aqueous electrolytes, based on the nontoxic ionomer LiPAA opens a range of possibilities in the search for new, cheap, safe, and environmentally benign electrolytes for LIB systems. The electrolyte containing 50 wt% LiPAA is a leak-free and dimensionally stable gel electrolyte that exhibits a high conductivity as well as a “water-in-ionomer” behavior, with a particularly noticeable enlargement of the ESW. It allows the design of environmentally friendly battery cell chemistries, free of nickel, cobalt, and fluorine, which can be operated up to 2.7 V with an initial energy density of 124 Wh kg^−1^ at the material level. It is worth mentioning that, if the most recent NiMH batteries have a specific energy of ca. 100 Wh kg^−1^, they could only store 54 Wh kg^−1^ at their commercial release^37^ and the earliest Li-ion batteries could only deliver 80 Wh kg^−1^ (vs. 250 Wh kg^−1^ for the most recent ones)^38^. Thus, the electrolyte concept developed in this study represents a step forward to a truly sustainable and nontoxic aqueous Li-ion battery with high energy density.

## Methods

### Electrode materials

**The cathode materials** (spinel LiMn_2_O_4_ and LiNi_0.5_Mn_1.5_O_4_) were purchased from Shanshan Technology Corporation and used without further purification. LiTi_2_(PO_4_)_3_ was synthesized by a phytic acid assisted solid-state method. In total, 17 mL tetrabutyl titanate (Ti(OC_4_H_9_)_4_, TBT) was first added to 500 mL of mixed butyl/ethanol (3:7 in volume ratio) solution under constant stirring to form white suspension. After stirring for 0.5 h, 3.12 g of lithium dihydrogen phosphate (LiH_2_PO_4_) which was dissolved in 10 mL of distilled water and 9.25 mL of physic acid (50 wt% in water) were added to the solution. The mixed solution was further stirred at room temperature for 4 h. The collected precipitate was washed with ethanol and distilled water, followed by drying at 80 °C to form the precursor. The LTP/C composite was obtained by heating the as-prepared precursor at 750 °C for 4 h under argon atmosphere. In a typical sythesis process of TiO_2_, 120 mL of ethanol solution containing 20 g of tetrabutyl titanate and 1.2 g of acetic acid was dropwise added into 240 mL of water/ethanol solution (3:1 in volume ratio) containing 8 g of oxalic acid (OA) and 0.8 g of sodium dodecylbenzenesulfonate (SDBS) under stirring at room temperature. The resulting light brown slurry was further stirred for 3 h and additionally aged for 1 h. The precipitate was collected by centrifuge and repeatedly washed with ethanol and deionized water, and then dried at 80 °C. The obtained nanocomposite was further calcined at 600 °C for 5 h under air, resulting in the TiO_2_ product. The morphologies and structures of these materials are confirmed by SEM and XRD, shown in Supplementary Figs. 10 and 11.

### Aqueous electrolyte

The lithium salt of poly(acrylic acid) was prepared by equilibrating the linear polymer (200 ml, 30% in water solution, Mw = 250,000) with dilute lithium hydroxide solution (0.05 mol L^−1^). Until the pH value became neutral, the neutral solution was kept constant stirring for 24 h. The functionalized polymer was obtained after all the water was evaporated at 80 °C. The electrolytes were then prepared by adding appropriate amounts of deionized water.

### Electrode material characterization

The crystal structure of the prepared materials was characterized by X-ray diffraction (XRD) on a Bruker D8 Advance (Bruker) diffractometer with Cu K_α_ radiation (1.54 Å) at room temperature. The pattern was recorded in the 2θ range of 10–90° at a scan rate of 0.0197° per step and a count time per step of 1 s. The particle morphology was evaluated using field-emission scanning electron microscopy (FE-SEM, Zeiss Auriga). The SEM images and XRD patterns of LiMn2O4, LiNi_0.5_Mn_1.5_O4, LiTi_2_(PO_4_)_3_, and TiO_2_ materials are showed in Supplementary Figs. 9 and 10.

### Electrochemical measurements

The slurry to prepare the electrodes was obtained by mixing the active materials, conductive carbon (Super C65, Timcal), and a binder (polyvinylidene difluoride PVdF, Kynar® FLEX 2801, Arkema Group) in a weight ratio of 80:12.5:7.5, with *N*-methyl-2-pyrrolidone (NMP) as the processing solvent. The well-mixed slurry was coated on a Cu foil and dried at 80 °C overnight. After being punched into Ø 13 mm discs, the electrodes (of ca. 4–5 mg cm^−2^, with cathode capacities of ca. 80% of that of the anodes) were further pressed on Ø 12 mm steel/aluminum mesh. The Cu foil was then removed and the electrodes were dried for 12 h under vacuum at 100 °C. The electrochemical performance was evaluated with a Swaglok cell system. The cathode and anode were separated by glass fiber (Whatman GF/D) impregnated with 1 mL of the electrolytes. Galvanostatic cycling tests were carried out on MACCOR series 4000 battery testers at various current rates.

## Electronic supplementary material

Supplementary Information

## Data Availability

The data generated during the current study are available from the corresponding author on reasonable request.
