# Supplementary material for: Fluorine-free water-in-ionomer electrolytes for sustainable lithium-ion batteries
Source: Nat Commun. 2018 Dec 14;9:5320. doi: 10.1038/s41467-018-07331-6 (PMC6294254; doi:10.1038/s41467-018-07331-6)
Supplement: Supplementary file 1 — Supplementary Information [file 41467_2018_7331_MOESM1_ESM.pdf]

Supplementary Materials for:

Fluorine-free *water-in-ionomer* electrolytes for sustainable lithium-ion batteries

Xin He et al.

Supplementary Figures

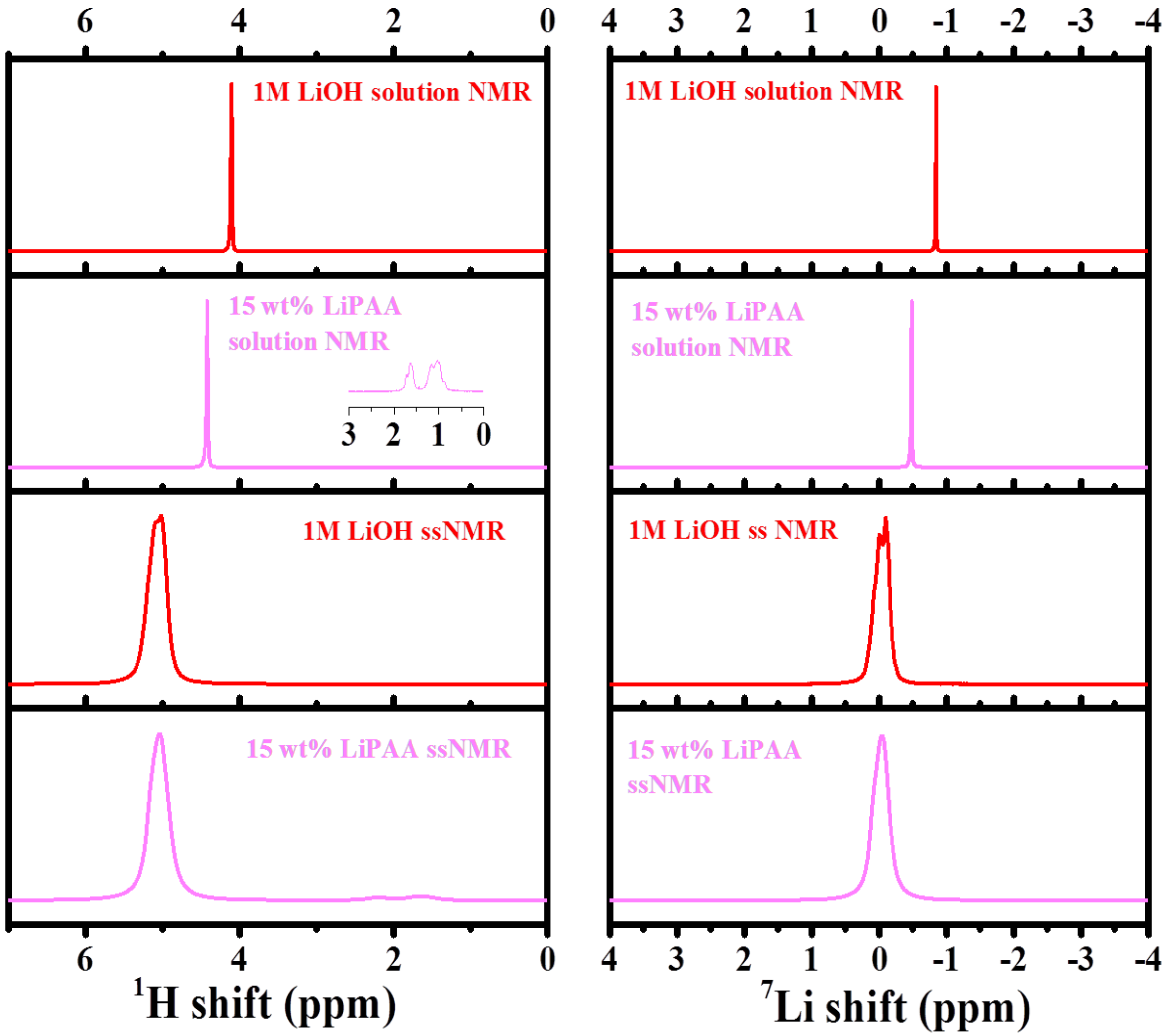

Supplementary Fig. 1. Comparison between the solution and solid state (MAS) ^{1}H and ^{7}Li NMR spectra of 15 wt% LiPAA electrolyte and 1M LiOH aqueous solutions.

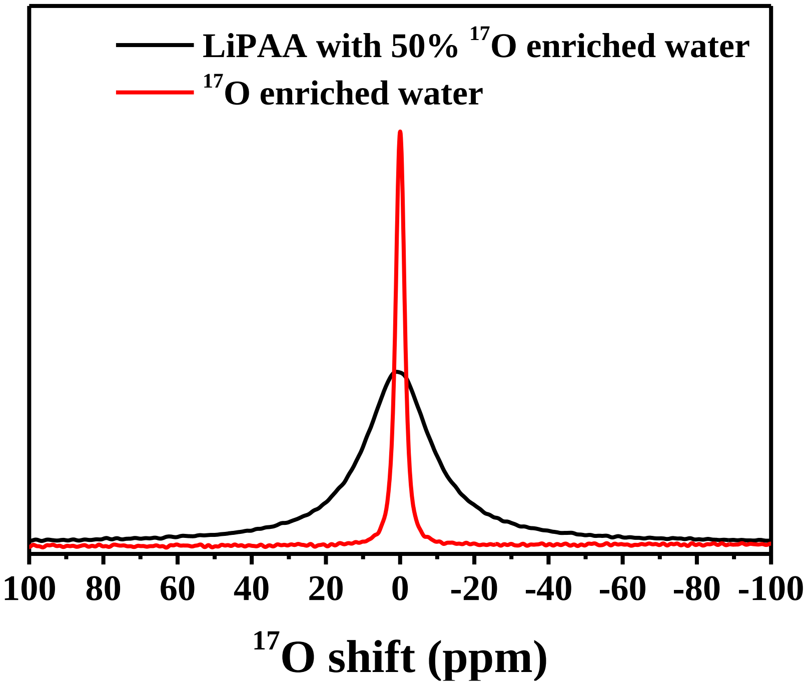

Supplementary Fig. 2. ^{17}O MAS NMR spectrum of the 50 wt% LiPAA electrolyte spinning at 14 kHz and ^{17}O static NMR spectrum of $H_2{}^{17}O$.

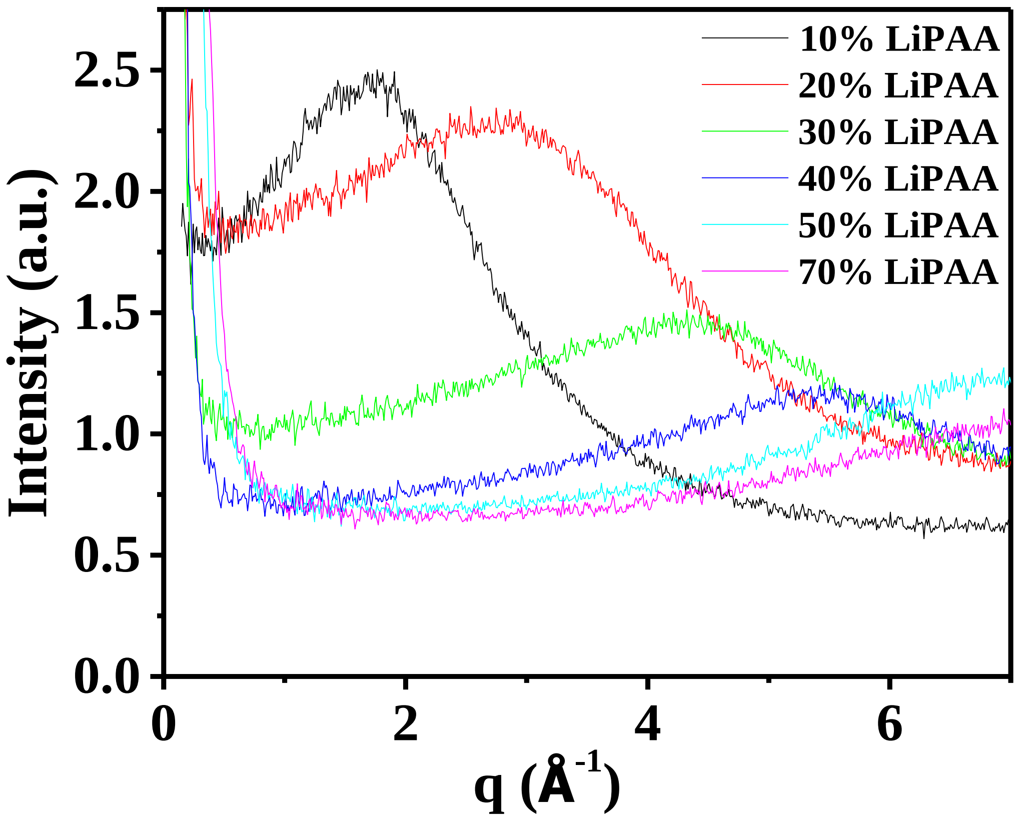

Supplementary Fig. 3. SAXS spectra obtained for the electrolytes from 10% to 70% LiPAA

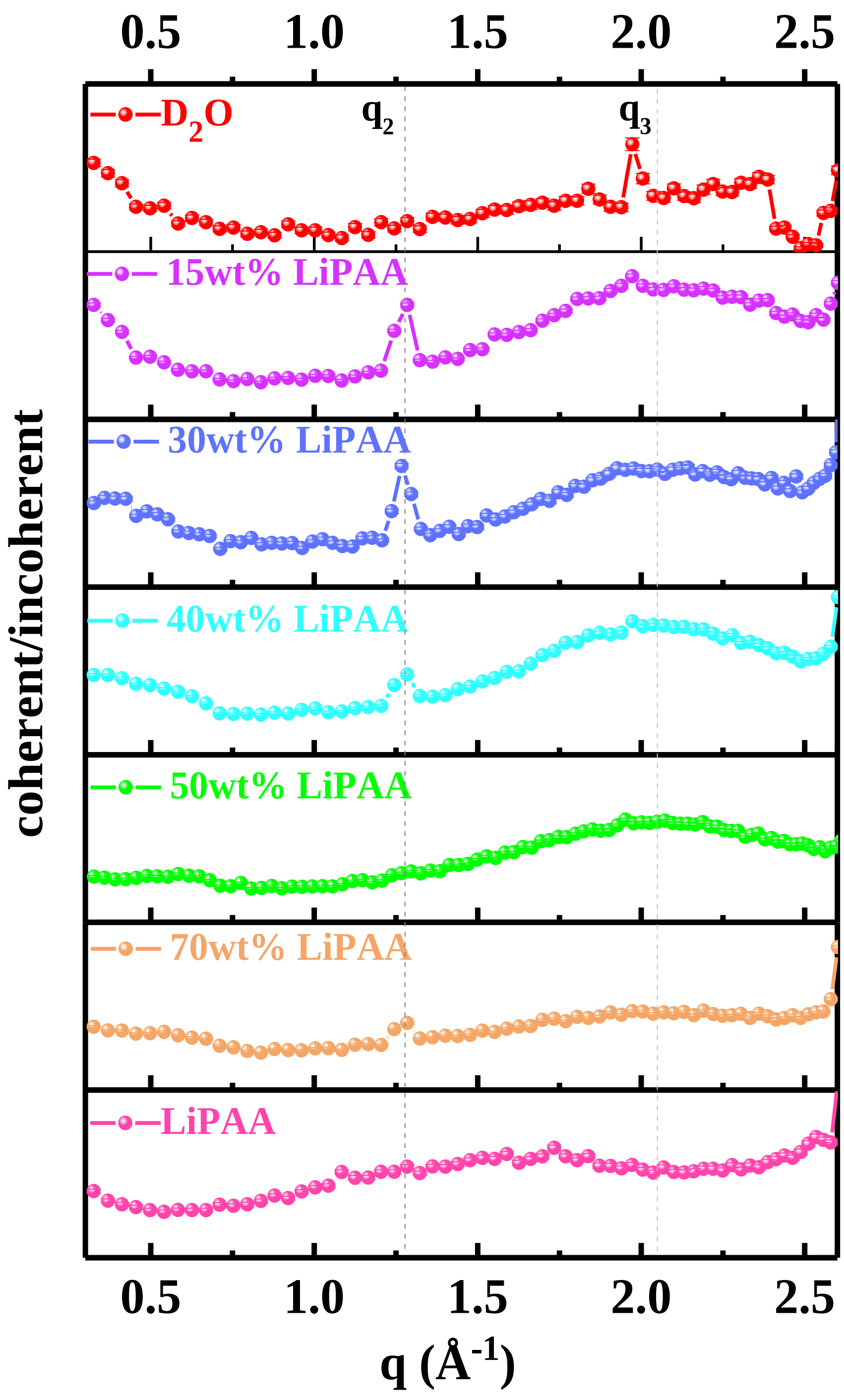

Supplementary Fig. 4. Ratios of coherent to incoherent scattering from polymer samples with different LiPAA concentrations in D_2O electrolyte.

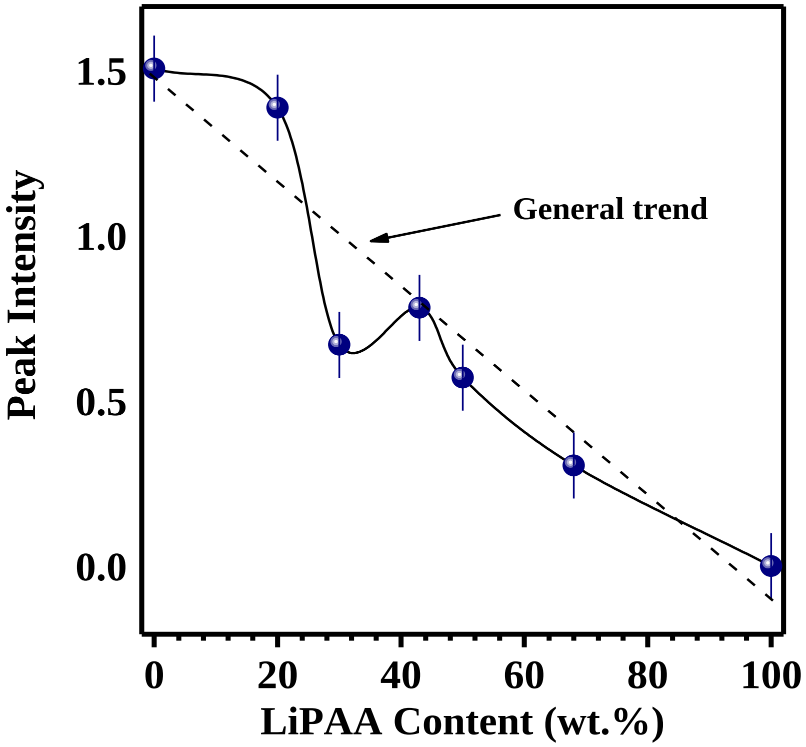

Supplementary Fig. 5. Intensity of the broad peak at 2.1$Å^{-1}$ in DNS spectrum as a function of the LiPAA content in D_2O electrolyte.

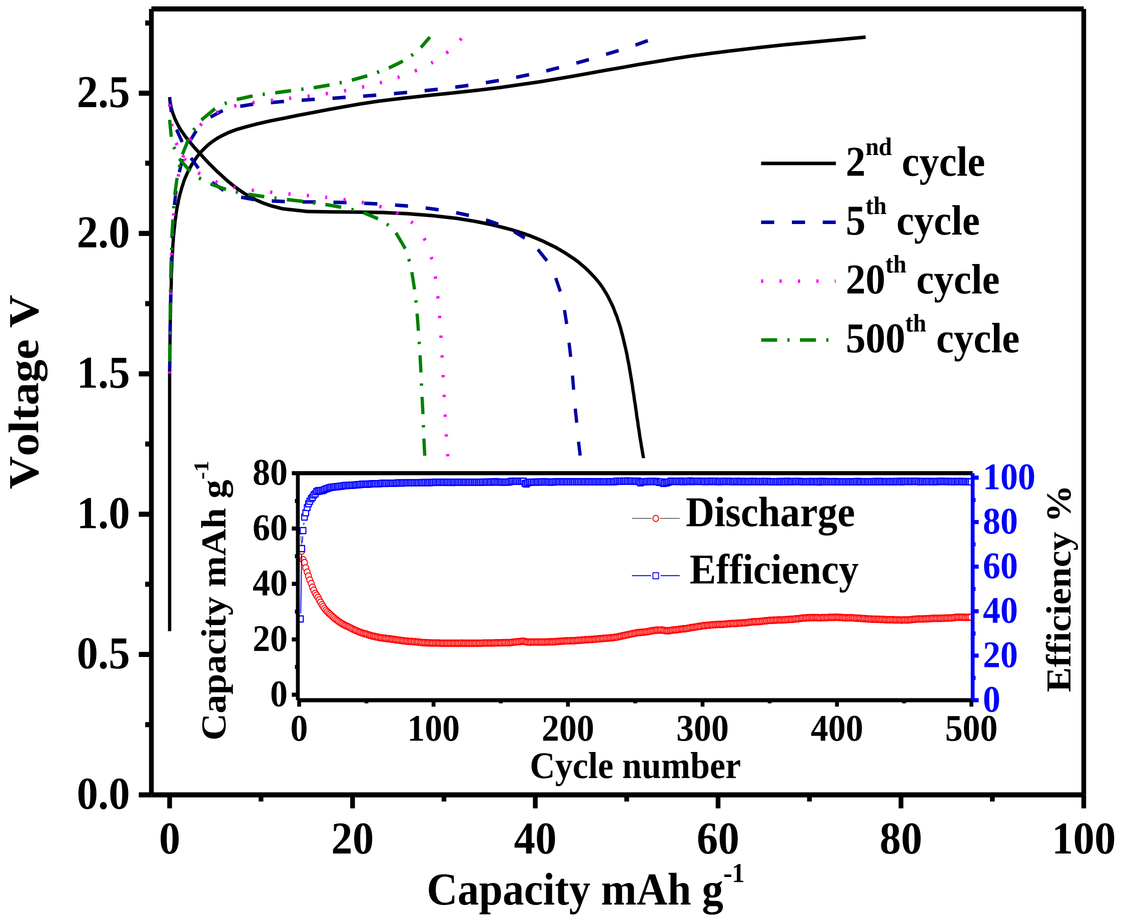

Supplementary Fig. 6. Charge-discharge voltage profiles (2nd, 5th, 20th and 500th cycles) of a $TiO_2/LiMn_2O_4$ cell at 5C in the voltage range of 1.2-2.7 V (based on $LiMn_2O_4$ and TiO_2 at 1:0.8 by weight in the 50 wt% LiPAA electrolyte). The current collector for $LiMn_2O_4$ was a SS mesh, and for TiO_2 an Al mesh. The cell capacity was calculated based on the total weight of the positive and negative active materials. The insert shows the discharge capacity and coulombic efficiency for 500 cycles.

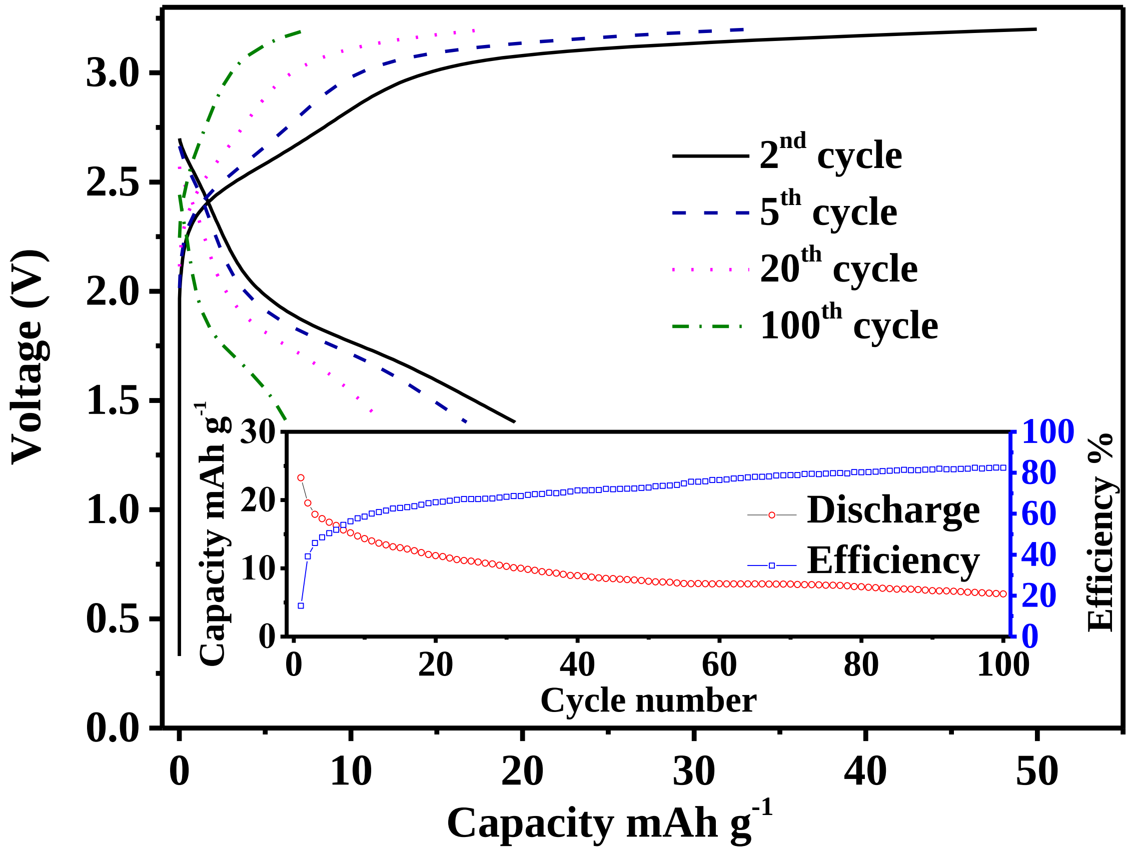

Supplementary Fig. 7. Charge-discharge voltage profiles (2nd, 5th, 20th and 500th cycles) of a $TiO_2/LiNi_{0.5}Mn_{1.5}O_4$ cell at 5C in the voltage range of 1.4-3.2 V (based on $LiNi_{0.5}Mn_{1.5}O_4$ and TiO_2 at 1:0.8 by weight in the 50 wt% LiPAA electrolyte). The current collectors were Al mesh for both electrodes. The cell capacity was calculated on the basis of the total weight of the positive and negative active materials. The inset figure shows the discharge capacity and coulombic efficiency for 100 cycles.

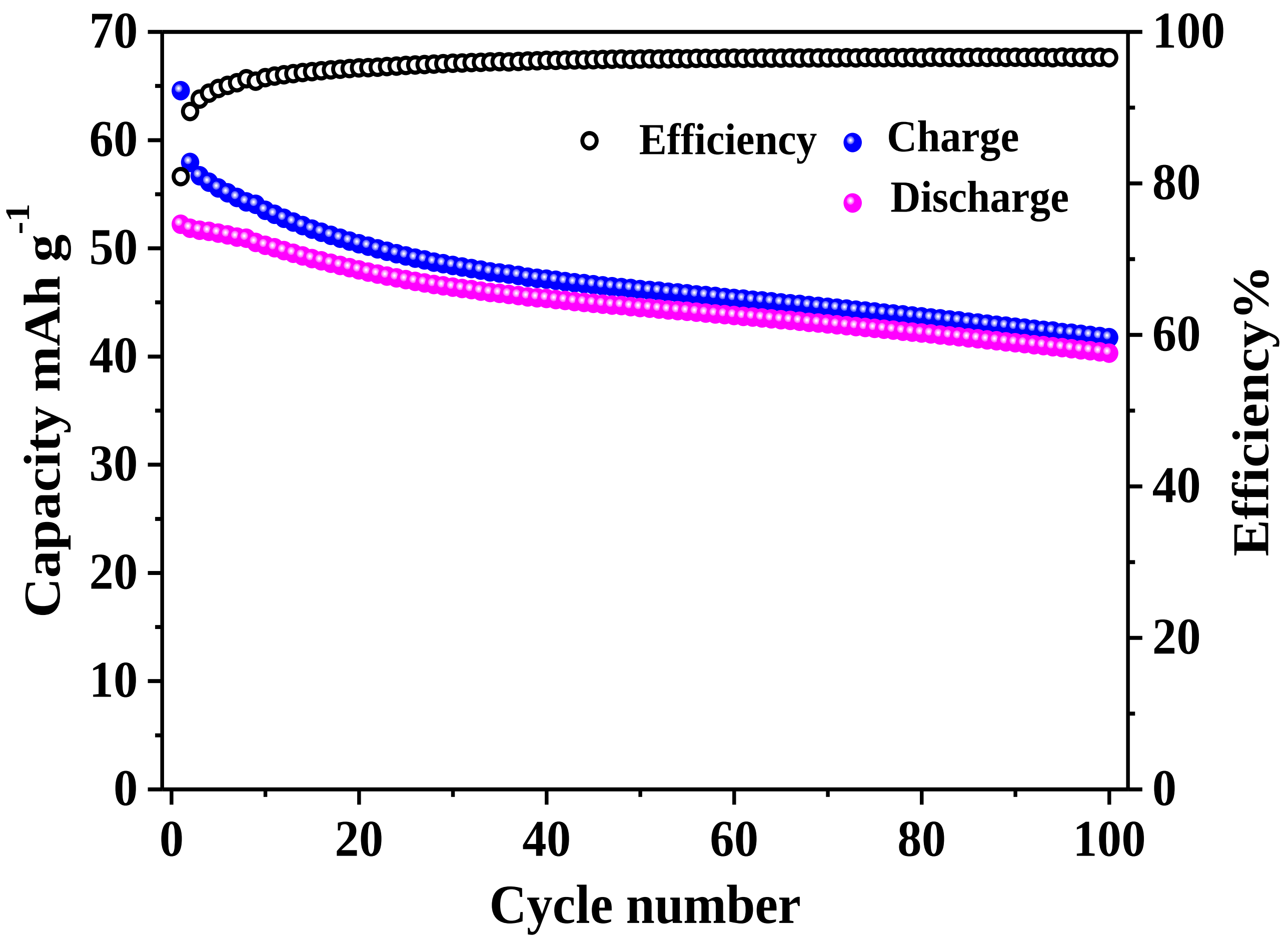

Supplementary Fig. 8. Specific capacity of a $LiTi_2(PO_4)_3/LiMn_2O_4$ full cell at 0.5 C in the voltage range from 0.8-2.0 V with 40 wt% LiPAA electrolyte for 100 cycles. (The full cell capacity is calculated based on $LiTi_2(PO_4)_3$ and $LiMn_2O_4$ at 1:0.8 by weight.

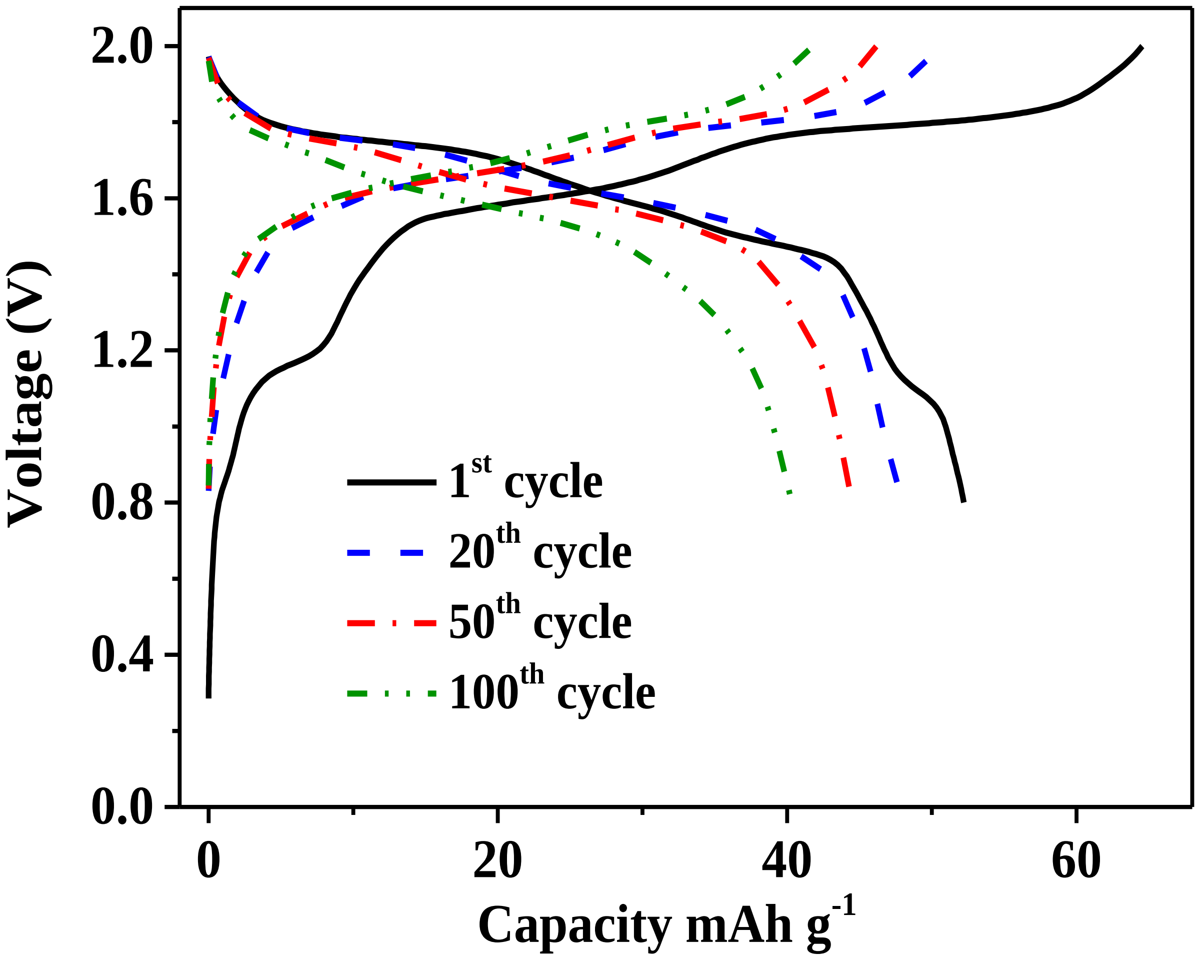

Supplementary Fig. 9. Charge-discharge voltage profiles (1nd, 20th, 50th and 100th cycles) of $LiTi_2(PO_4)_3/LiMn_2O_4$ full cell at 0.5C in the voltage range from 0.8-2.0 V in the 40 wt% LiPAA electrolyte (Specific capacity was calculated based on $LiTi_2(PO_4)_3$ and $LiMn_2O_4$ at 1:0.8 by weight).

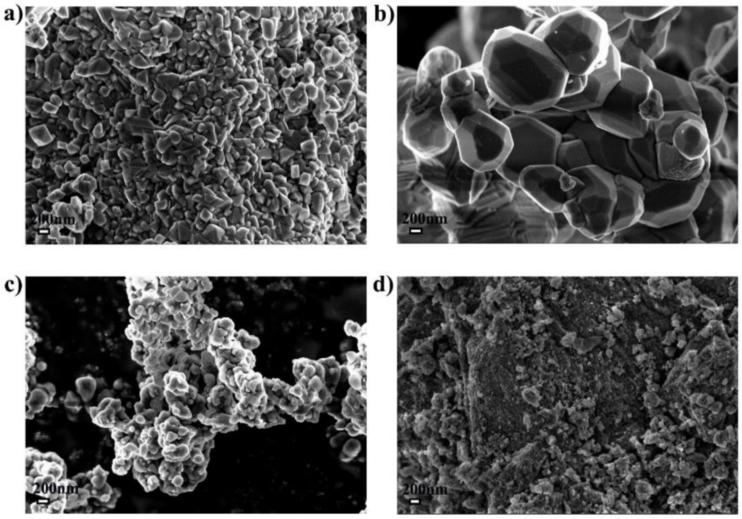

Supplementary Fig. 10. SEM images of (a) $LiMn_2O_4$, (b) $LiNi_{0.5}Mn_{1.5}O_4$, (c) $LiTi_2(PO_4)_3$ and (d) TiO_2 materials.

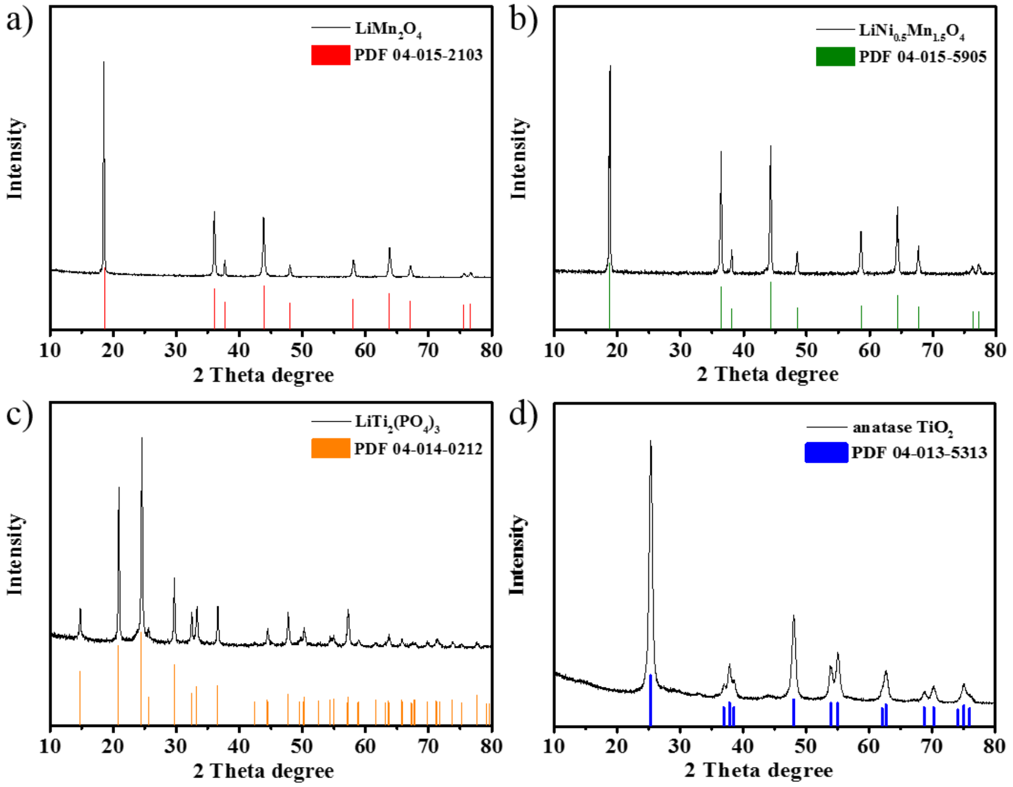

Supplementary Fig. 11. XRD patterns of LiMn2O4 (a), $LiNi_{0.5}Mn_{1.5}O4$ (b), $LiTi_2(PO_4)_3$ (c) and TiO_2 (d) materials.

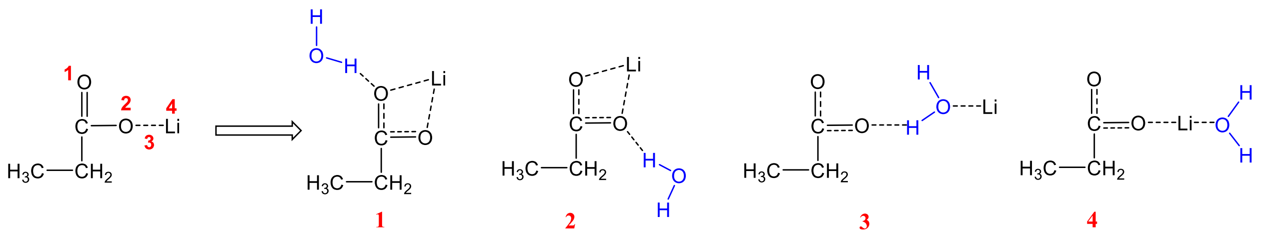

Supplementary Fig. 12. Scheme of adding water molecules to each of the four sites of a LiPAA monomer.

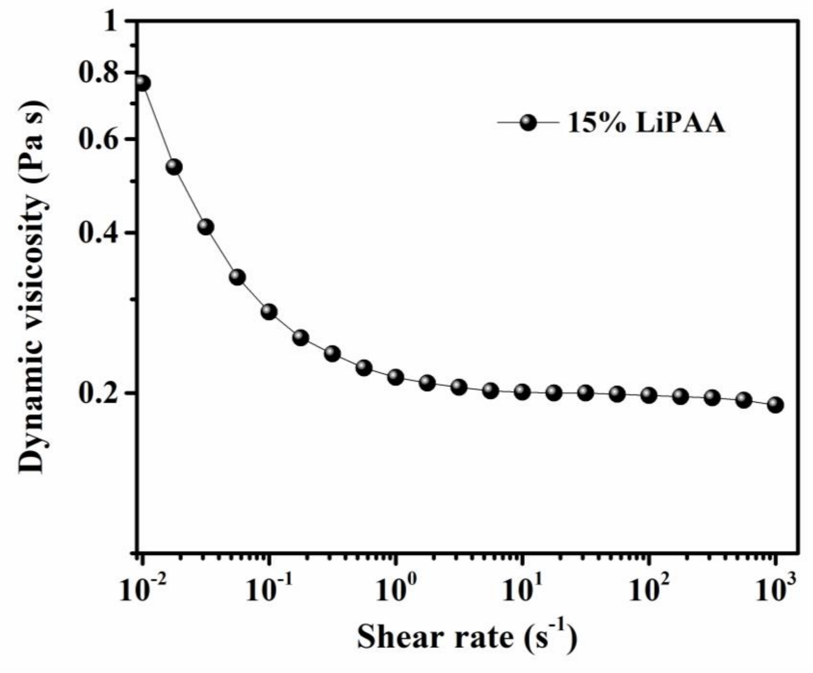

Supplementary Fig. 13. Evolution of the dynamic viscosity of the 15% LiPAA electrolyte with shear rate.

LiPAA

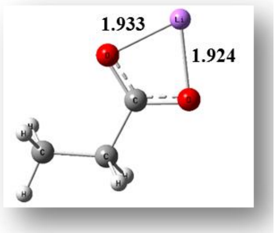

LiPAA(H_2O)

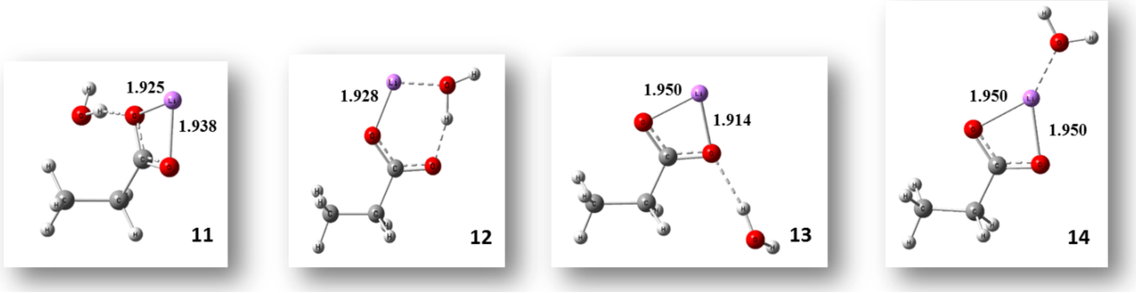

LiPAA(H_2O)$_2$

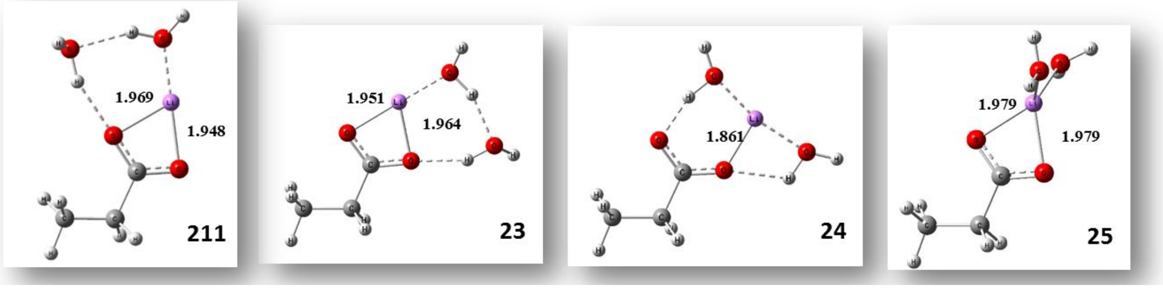

LiPAA(H_2O)$_3$

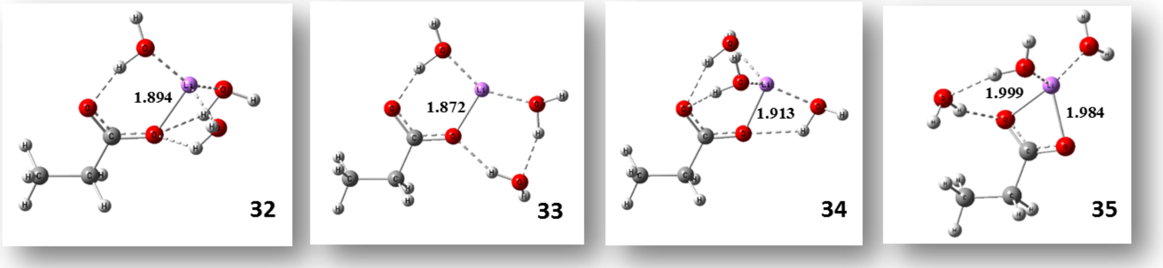

LiPAA(H_2O)$_4$

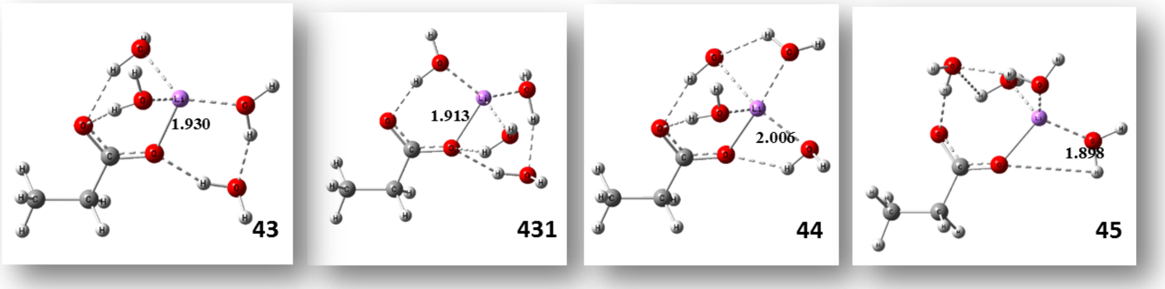

LiPAA(H_2O)$_5$

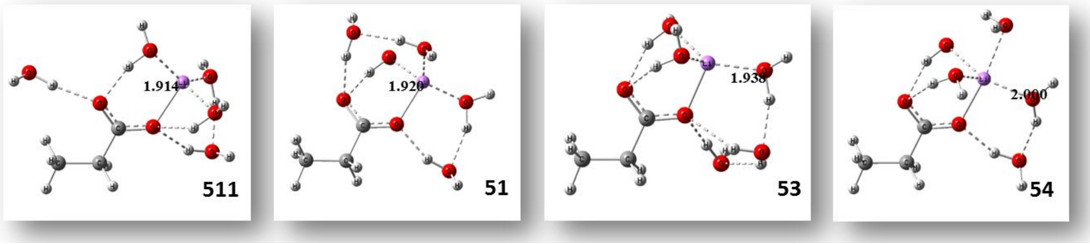

LiPAA(H_2O)$_6$

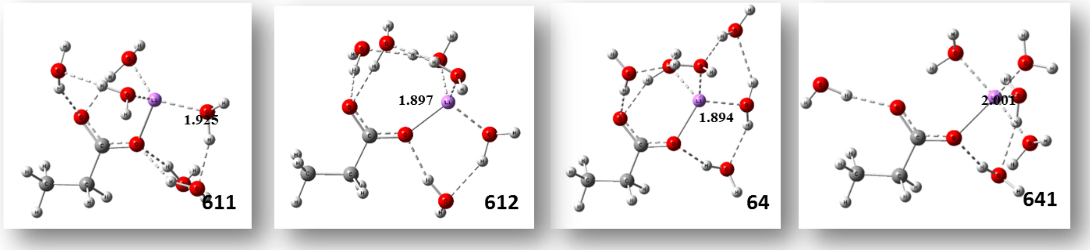

LiPAA(H_2O)$_7$

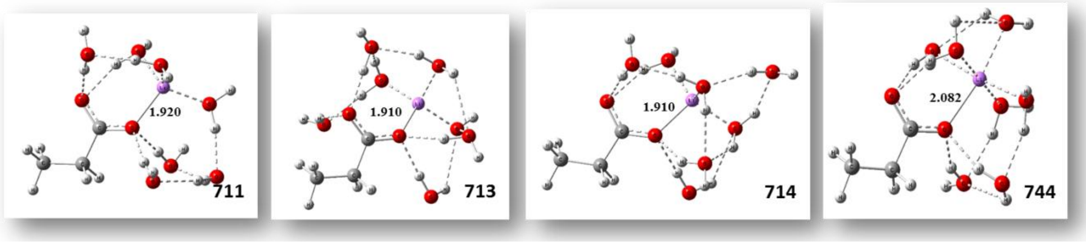

LiPAA(H_2O)$_8$

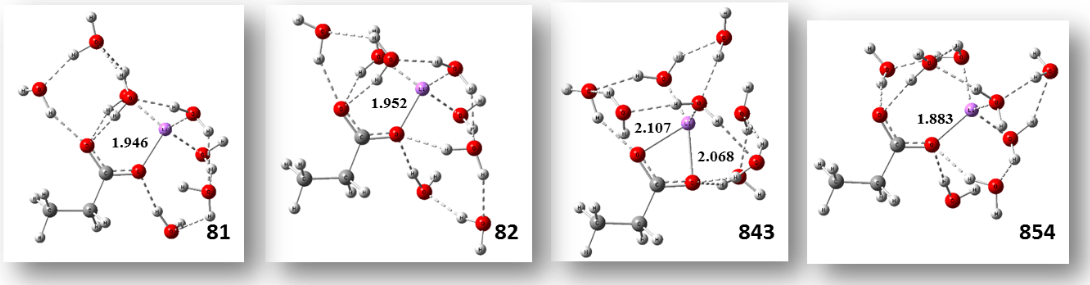

Supplementary Fig. 14. Selected structures and Li^+-O bond lengths (unit: Å) of LiPAA(H_2O)$_n$ (n = 0-8) clusters as calculated by M08-HX/MG3S/SMD.

Supplementary Tables

Supplementary Table 1. Electrolytes prepared and corresponding ratio of the number of H_2O or D_2O molecules to the number of Li^+ cations.

LiPAA wt %/name of electrolyte	H_2O per Li^+	D_2O per Li^+
84 wt% LiPAA	0.83	0.74
70 wt% LiPAA	1.86	1.67
50 wt% LiPAA	4.33	3.0
40 wt% LiPAA	6.5	5.85
30 wt% LiPAA	10.1	9.1
15 wt% LiPAA	24.6	22.1
10 wt% LiPAA	39	35.1

Supplementary Table 2. Vogel Tammann Fulcher (VTF) parameters for conductivity and R^2 values for the linear fit

wt% LiPAA	σ_0 /S cm^{-1}	E_a /J mol^{-1}	T_0 /K	R^2
**70**	4857.8	9093.5	174.9	0.9999210
**50**	1983.0	6066.0	166.0	0.9998735
**40**	1415.1	5246.1	169.6	0.9999649
**30**	908.94	4075.5	167.3	0.9999723

Supplementary Table 3. Different peak widths observed for the 15 wt% LiPAA electrolyte and 1M LiOH aqueous solution, acquired with solution and solid-state NMR spectrometers.

		Peak width (Hz)	
		Solution NMR	Solid-state NMR
**15 wt% LiPAA**	^{1}H (water)	10.4	132.8
	^{7}Li	2.0	51.8
**1M LiOH**	^{1}H (water)	8.3	123.7
	^{7}Li	1.5	49.3

Supplementary Table 4. Relative energies of LiPAA(H_2O)$_n$ (n = 1-8) clusters as calculated by M08-HX/MG3S/SMD. Note that $G_{\mathrm{bind}}°$ is defined in supplementary eq. 4, and $U_{\mathrm{e,bind}}°$ and $U_{0,\mathrm{bind}}°$ are defined by supplementary eq. (6 & 7). The results for the lowest-energy conformer for each n is bold, and $\Delta U_{\mathrm{e,bind}}°$, $\Delta U_{0,\mathrm{bind}}°$, and $\Delta G_{\mathrm{bind}}°$ of the other conformers with that n are given relative to this conformer.

	$U_\mathrm{e}°$ (hartree)	$U_{\mathrm{e,bind}}°$ (kcal/mol)	$\Delta U_{\mathrm{e,bind}}°$ (kcal/mol)	$U_0°$ (hartree)	$U_{0,\mathrm{bind}}°$ (kcal/mol)	$\Delta U_{0,\mathrm{bind}}°$ (kcal/mol)	$G°$ (hartree)	$G_{\mathrm{bind}}°$ (kcal/mol)	$\Delta G_{\mathrm{bind}}°$ (kcal/mol)
H_2O	-76.43918	/	/	-76.41805	/	/	-76.43600	/	/
LiPAA	-275.42523	/	/	-275.34837	/	/	-275.37834	/	/
**11**	-351.87168	4.6	-12.4	-351.76980	2.1	-12.7	-351.80489	-6.0	-12.7
**12**	-351.88321	11.8	-5.2	-351.78163	9.5	-5.3	-351.81562	0.8	-5.9
**13**	-351.87244	5.0	-12.0	-351.77118	3.0	-11.8	-351.80586	-5.3	-12.0
**14**	**-351.89150**	**17.0**	**0.0**	**-351.79005**	**14.8**	**0.0**	**-351.82502**	**6.7**	**0.0**
**211**	-428.33921	5.4	-7.3	-428.21341	3.3	-8.1	-428.25337	-4.8	-8.4
**23**	-428.33956	5.6	-7.1	-428.21319	3.2	-8.2	-428.25183	-5.8	-9.3
**24**	-428.34756	10.6	-2.1	-428.22167	8.5	-2.9	-428.26148	0.3	-3.3
**25**	**-428.35083**	**12.6**	**0.0**	**-428.22625**	**11.4**	**0.0**	**-428.26668**	**3.6**	**0.0**
**32**	**-504.80417**	**8.9**	**0.0**	**-504.65484**	**6.6**	**0.0**	**-504.69981**	**-1.8**	**0.0**
**33**	-504.79764	4.8	-4.1	-504.64711	1.8	-4.8	-504.69046	-7.7	-5.9
**34**	-504.80389	8.7	-0.2	-504.65391	6.0	-0.6	-504.69744	-3.3	-1.5
**35**	-504.80048	6.6	-2.3	-504.65110	4.3	-2.3	-504.69619	-4.1	-2.3
**431**	-581.25453	7.0	-0.5	-581.0799	4.4	-0.5	-581.12596	-6.2	-0.8
**43**	-581.25464	7.1	-0.4	-581.07977	4.3	-0.5	-581.12622	-6.0	-0.6
**44**	-581.25361	6.4	-1.1	-581.07964	4.2	-0.6	-581.12687	-5.6	-0.2
**45**	**-581.25529**	**7.5**	**0.0**	**-581.08059**	**4.8**	**0.0**	**-581.12717**	**-5.4**	**0.0**
**511**	-657.70158	4.5	-2.9	-657.50344	3.0	-2.3	-657.55479	-5.3	-2.4
**51**	**-657.70628**	**7.4**	**0.0**	**-657.50708**	**5.3**	**0.0**	**-657.55864**	**-2.8**	**0.0**
**53**	-657.70136	4.3	-3.1	-657.50264	2.5	-2.8	-657.55250	-6.7	-3.9
**54**	-657.70427	6.2	-1.3	-657.50578	4.5	-0.8	-657.55479	-5.3	-2.4
**611**	-734.15330	4.9	-1.2	-733.92960	2.8	-1.5	-733.98175	-8.1	-1.7
**612**	**-734.15519**	**6.1**	**0.0**	**-733.93204**	**4.3**	**0.0**	**-733.98442**	**-6.4**	**0.0**
**641**	-734.15068	3.3	-2.8	-733.92881	2.3	-2.0	-733.98302	-7.3	-0.9
**64**	-734.15262	4.5	-1.6	-733.92916	2.5	-1.8	-733.98254	-7.6	-1.2
**711**	-810.60257	5.2	-1.2	-810.35427	2.6	-2.1	-810.41034	-6.3	-1.4
**713**	-810.60258	5.2	-1.2	-810.35502	3.1	-1.7	-810.41092	-6.0	-1.1
**714**	-810.60117	4.3	-2.1	-810.35190	1.1	-3.6	-810.40605	-9.0	-4.1
**744**	**-810.60452**	**6.4**	**0.0**	**-810.35769**	**4.8**	**0.0**	**-810.41261**	**-4.9**	**0.0**
**81**	**-887.05134**	**4.8**	**0.0**	**-886.77973**	**2.5**	**0.0**	**-886.84209**	**-4.1**	**0.0**
**82**	-887.0489	3.2	-1.6	-886.77676	0.6	-1.9	-886.83683	-7.4	-3.3
**843**	-887.05119	4.7	-0.1	-886.77938	2.3	-0.2	-886.83990	-5.5	-1.4
**854**	-887.04991	3.9	-0.9	-886.77828	1.6	-0.9	-886.83967	-5.6	-1.5

Supplementary Table 5. ^{1}H (PAA^-) and ^{7}Li (Li^+) diffusion coefficients and calculated Li^+ transference number

Concentration of LiPAA	Diffusion coefficient of PAA^- ($m^2 s^{-1}$)	Diffusion coefficient of Li^+ ($m^2 s^{-1}$)	Lithium transference number
60%	---	$8.09\ 10^{-13}$	
50%	$1.75\ 10^{-12}$	$5.99\ 10^{-12}$	0.77
40%	$1.84\ 10^{-11}$	$5.17\ 10^{-11}$	0.74
30%	$2.85\ 10^{-11}$	$8.28\ 10^{-11}$	0.74
15%	$9.79\ 10^{-11}$	$1.80\ 10^{-10}$	0.65

Supplementary Notes

Supplementary Note 1. Rheological properties

As can be seen in Fig. 1b, the 30 % LiPAA and the 40 % LiPAA gels exhibit rather similar behavior, with their loss modulus higher than their storage modulus (i.e. a tan δ lower than 1) at low shear strain and above 1 at higher shear strain. Moreover, values close to each other are obtained for the 30 % and 40 % electrolytes and the storage modulus clearly decreases at higher strains. This behavior is less marked for the 40% LiPAA gel already. For the 50 % electrolyte, there is a clear jump in loss and storage modulus and tan δ is above 1 on the whole investigated range, which indicates a more 'elastic' behavior. Moreover, the storage modulus increases slightly with the strain contrary to all the other gels.
The 70 % gel shows a further increases for both modulus. However, the loss modulus is also, in this case, above the storage modulus at low deformations, although both converge to the same values at higher deformation, and decrease. Thus, it seems that the 50 % LiPAA gel exhibits a more elastic behavior and peculiar mechanical properties as compared to the gels with both lower and higher LiPAA contents, which might correspond to different local structure.
The viscosity of the 15 % LiPAA electrolyte was measured in rotation mode with the same cone geometry and the results are shown in Supplementary Fig. 13. As can be seen, the solution exhibits a non-newtonian behavior, with clear shear thinning with increasing sheer rate, which also indicates a structured electrolyte in this case.

Supplementary Note 2. Determination of Vogel-Tammann-Fulcher (VTF) paramaters

The conductivity curves were fitted with the following version of the Vogel-Tamman-Fulcher (VTF) equation to extract the parameters σ_0 and Ea (of the same dimension as the Arrhenius' activation energy and sometimes considered as such [1]) and T_0

$$\sigma = \sigma_0 e^{-\frac{E_a}{R(T-T_0)}} \tag{1}$$

The conductivity curves were first linearized by plotting

$$\ln(\sigma) = f\left(\frac{1}{T-T_0}\right) \tag{2}$$

The T_0 values were varied by 0.1 K increments until reaching a maximum for the R^2 of the linear fit. The parameters as well as the R^2 values are reported in Supplementary Table 2.

As can be seen, as the concentration of LiPAA decreases, so does σ_0 (which depends on the concentration of mobile charged species participating to conduction). The T_0 value, which usually scales with Tg and the mobility of the electrolytes are in a rather narrow range, with only a marked decrease from 70% LiPAA to 50%. The values are then surprisingly similar (given the differences in mechanical properties (see Fig. 1b), which usually translate into differences in T_gs and T_0 for

homogeneous gels). It is thus likely that the mechanical properties of the gels are linked to the local structure of the polymer domains rather than to the mobility within the conductive domains. Ea and σ_0 increase rather linearly with the LiPAA content, in accordance with the increasing density of charge carrier (for σ_0) and a more difficult movement of ions.

Supplementary Note 3. Determination of Li^+ transference numbers

The ^{1}H and ^{7}Li diffusion coefficients (D_H and D_{Li}) of LiPAA samples were measured at 293 K using pulsed-field-gradient NMR (PFG-NMR) with a diffBB probe, employing a stimulated echo pulse sequence (ledgp2s), on a 600 MHz Bruker NMR spectrometer with a permanent field strength of 14.1 T. The results are given in Supplementary Table 5. The maximum gradient strength was 20 T/m. Lithium transference number T^+ was obtained by the ratio of $D_{Li}/(D_{Li} + D_H)$. The diffusion coefficient was determined by measuring the decay of the signal intensity *I* in dependence of the gradient strength *g*

$$I = I_0 \exp\left(-D\gamma^2 g^2 \delta^2 \left(\Delta - \frac{\delta}{3}\right)\right) \tag{9}$$

Where *I* is the observed intensity, I_0 the reference intensity, D the diffusion coefficient, γ is the gyromagnetic ratio of the observed nucleus, *g* the gradient pulse length, δ the length of the gradient, and Δ the diffusion time. As can be seen, the transference number are rather high (well above 0.5), especially for the gels, in which the polymer movement is more limited (T^+ = 0.74-0.77). Further increase would require either crosslinking of the polymer or using block copolymer (i.e. adding a hydrophobic block) to obtain phase separation (rather than local structuration), and complete immobilization of the polymer chains.

Supplementary Note 4. Details of Quantum Mechanical Modeling

For modeling we considered a monomer of LiPAA and up to 8 water molecules. We carried out full geometry optimizations for LiPAA(H_2O)n (n = 0–8) by Kohn-Sham density functional theory with the M08-HX global-hybrid meta-GGA density functional[2] and the MG3S basis set[3]. Vibrational frequencies were scaled by a factor of 0.973 to improve the accuracy of the calculated zero point energy[4]. All the calculations included the effect of the surrounding environment by the SMD solvation model[5]. Because the dielectric constant ε of the environment in which the cluster model is located should be between that of water and that of the superabsorbent LiPAA polymer, we used 1,2-ethanediol (with ε = 40.245) as the solvent surrounding the LiPAA$(H_2O)_n$ (n = 0-8) complexes. Density functional integrals were computed using a grid of 974 angular points per shell and 99 radial shells. “Tight” convergence criteria were used in the optimization. All of the quantum mechanical calculations of LiPAA$(H_2O)_n$ (n = 0–8) were carried out with the *Gaussian 09* software package[6].

The partial atomic charges on LiPAA$(H_2O)_n$ (n = 0-8) were calculated using the CM5PAC package,[7] which utilizes Hirshfeld atomic charges [8] to obtain partial atomic charges by Charge Model 5 (CM5)[9].

The binding free energy of the nth water molecule in LiPAA$(H_2O)_n$ is defined as the standard-state Gibbs free energy change in the reaction

$$\text{LiPAA}(H_2O)_n(\text{solv}) \rightarrow \text{LiPAA}(H_2O)_{n-1}(\text{solv}) + H_2O(\text{solv}) \quad (3)$$

where “(solv)” denotes that the species is dissolved in 1,2-ethanediol. This free energy change was calculated using the equation

$$G_{\text{bind}}° = G°[\text{LiPAA}(H_2O)_{n-1}(\text{solv})] + G°[H_2O(\text{solv})] - G°[\text{LiPAA}(H_2O)_n(\text{solv})] \quad (4)$$

where the Gibbs free energy is [10],

$$G°[X] = U_e°[X] + G_{\text{int}}[X] \quad (5)$$

and where $U_e°[X]$ is the solution-phase potential energy species X, defined as the equilibrium value on the potential of mean force (also known as the free energy surface)[54] including the standard-state free energy of solvation, ZPE[X] is the local zero-point vibrational energy, and $G_{\text{int}}[X]$ is the vibrational–rotational free energy of species X with local zero of energy at $U_e°[X]$ (where X is one of the species in eq. (3))[10]. In the article and in the rest of this supplementary material we refer to $U_e°$ values simply as energies as a substitute for the long phrase “equilibrium potential energy of mean force.” The superscript “°” denotes the standard state; all free energies are standard-state free energies with a solution-phase standard state of 1 mol L^{-1}. For each complex, the conformer with the lowest energy in 1,2-ethanediol was chosen to calculate the binding free energies. We also calculated binding energies by

$$U_{e,\text{bind}}° = U_e°[\text{LiPAA}(H_2O)_{n-1}] + U_e°[H_2O] - U_e°[\text{LiPAA}(H_2O)_n] \quad (6)$$

$$U_{0,\text{bind}}° = U_0°[\text{LiPAA}(H_2O)_{n-1}] + U_0°[H_2O] - U_0°[\text{LiPAA}(H_2O)_n] \quad (7)$$

where

$$U_0°[X] = U_e°[X] + \varepsilon^G[X] \quad (8)$$

and where $\varepsilon^G[X]$ is the zero point energy. Note that $G_{bind}°$, $U_{e,bind}°$, and $U_{0,bind}°$ are each defined such that a positive value indicates binding, i.e., indicates that binding is exergonic (for $G_{bind}°$) or exoergic (for $U_{e,bind}°$ and $U_{0,bind}°$). The calculated binding energies and binding free energies are given in Supplementary Table 4.

There are four sites per carboxylate group in LiPAA that can bind to water molecules, as shown in Supplementary Fig. 12. Thus there are at least four conformers of LiPAA(H_2O). The conformations of LiPAA$(H_2O)_2$ isomers were obtained by inserting a water molecule at four sites in the lowest energy structure of LiPAA(H_2O). Similarly the structures of LiPAA$(H_2O)_n$ were obtained by starting with the lowest energy structure of LiPAA$(H_2O)_{n-1}$. The isomers of LiPAA$(H_2O)_n$ (n = 1–8) obtained by this method are only a subset of the myriad of conformations existing in solution, and they need not contain the lowest-energy conformations for each n, but we use them as representative low-energy structures. As mentioned above, the lowest-energy ones for each n were used to calculate binding energies and binding free energies. By adopting this systematic method, we are able to gain insight into the effect of solvent in the LiPAA electrolyte.

The first and second water bind to the Li^+ cation and do not form hydrogen bonds with the carboxylate oxygens. Their bindings increase the Li-O bond length in the LiPAA unit. The third water binds to Li^+ and in this structure (structure **32** in Supplementary Fig. 14, which names and shows all the structures), there are hydrogen bonds to both carboxylate oxygens. The most stable conformation of LiPAA$(H_2O)_4$ (structure **45**) has Li^+ combining with three water molecules, and O1 from LiPAA binding one water molecule; in this way a hydrogen bonding network is formed among the four water molecules. The conformation with four water molecules being bound by the lithium ion (structure **44**) has an energy 0.6 kcal mol^{-1} higher than three water molecules bound (structure **45**), but the free energy is only 0.2 kcal mol^{-1} higher. These calculation results show that Li^+ preferentially combines three waters in addition to its binding to O2 from LiPAA and thereby it has a four-coordinated conformation. The fifth water binds to the other oxygen of the carboxylate group. Starting with LiPAA$(H_2O)_6$, some of the isomers contain outer water molecules that are only indirectly connected to the LiPAA, such as **64**, **711**, and **714**. Outer water appears in all isomers of LiPAA$(H_2O)_8$, indicating that the LiPAA cluster is saturated and that outer waters finally form free water.

Supplementary Methods

Solid State NMR: ^1H, ^7Li and ^{17}O solid state NMR spectra were acquired on a Bruker AVIII-500 spectrometer. All the NMR spectra of LiPAA electrolytes were carried out in a 4 mm triple-resonance probe head with a spinning rate of 14 kHz. 1M LiOH solution and ^{17}O enriched water were measured under static condition. A rotor-synchronised Hahn-echo pulse sequence with 90° pulse length of 2.65, 2.95 and 5.05 μs and a recycle delay of 10, 30 and 5 s were used for ^1H, ^7Li

and ^{17}O, respectively. ^{1}H, ^{7}Li and ^{17}O shifts were referenced to adamantane powder (1.87 ppm), LiF powder (-1 ppm) and liquid $H_2{}^{17}O$ (0 ppm), respectively.

Solution NMR: ^{1}H and ^{7}Li NMR were measured on a Bruker Avance III HD 500MHz Smart Probe spectrometer with a 3 mm NMR tube filled with ~0.1 ml sample solution. 1M LiOH in H_2O and 15 wt % LiPAA in H_2O are prepared in a Ar-filled glovebox. ^{7}Li shifts was referenced to LiCl solution (0 ppm). No deuterated solvent was used here (and hence no locking was performed) so the ^{1}H shifts are not accurate and cannot be readily compared.

Rheological properties

The rheological behavior of the gels from 30% to 70% LiPAA was measured in oscillation mode with a MCR102 rheometer (Anton Paar), using a cone plate (Cp50-1, Diameter- 49.966mm) with a gap of 0.1 mm, at 20°C, with controlled shear strain (via deflection angle) at 10 rad s^{-1}.

Small angle X-ray Scattering (SAXS): SAXS measurements were carried out at the GALAXI diffractometer equipped with a Dectris Pilatus 1M detector and a BRUKER AXS Metaljet X-ray source at JCNS[11]. The applied wavelength and sample to detector distance are 1.34 Å and 831 mm, respectively. Samples were placed in borosilicate glass capillaries and each measurement counted 1200s.

Wide angle neutron scattering (WANS): Neutron scattering data at wide angles were collected with diffuse scattering neutron time-of-flight spectrometer (DNS) from Jülich Centre for Neutron Science (JCNS) at the FRM-2 in Garching[12]. A combination of large double-focusing PG (002) (d = 3.355 Å) monochromator and a highly efficient supermirror-based polarizer provide a polarized neutron flux of about 10^{7} n cm^{-2} s^{-1}. The coherent and incoherent scattering could be distinguished by polarization analysis of the scattered neutrons. We followed the analysis described in Supplementary Ref. 13.

Supplementary References

1. Diederichsen, K. M., Buss, H. G. & McCloskey, B. D. The Compensation Effect in the Vogel–Tammann–Fulcher (VTF) Equation for Polymer-Based Electrolytes. *Macromolecules* **50**, 3831–3840 (2017).
2. Zhao, Y. & Truhlar, D. G. Exploring the Limit of Accuracy of the Global Hybrid Meta Density Functional for Main-Group Thermochemistry, Kinetics, and Noncovalent Interactions. *J. Chem. Theory Comput.* **4**, 1849–1868 (2008).
3. Lynch, B. J., Zhao, Y. & Truhlar, D. G. Effectiveness of Diffuse Basis Functions for Calculating Relative Energies by Density Functional Theory. *J. Phys. Chem. A* **107**, 1384–1388 (2003).
4. Alecu, I. M., Zheng, J., Zhao, Y. & Truhlar, D. G. Computational Thermochemistry: Scale Factor Databases and Scale Factors for Vibrational Frequencies Obtained from Electronic Model Chemistries. *J. Chem. Theory Comput.* **6**, 2872–2887 (2010).
5. Marenich, A. V., Cramer, C. J. & Truhlar, D. G. Universal Solvation Model Based on Solute Electron Density and on a Continuum Model of the Solvent Defined by the Bulk Dielectric Constant and Atomic Surface Tensions. *J. Phys. Chem. B* **113**, 6378–6396 (2009).
6. M. J. Frisch, G. W. T., H. B. Schlegel, G. E. Scuseria, M. A. Robb, J. R. Cheeseman, G. Scalmani, V. Barone, G. A. Petersson, H. Nakatsuji, X. Li, M. Caricato, A. Marenich, J. Bloino, B. G. Janesko, R. Gomperts, B. Mennucci, H. P. Hratchian, J. V. Ortiz, A. F. Izmaylov, J. L. Sonnenberg, D. Williams-Young, F. Ding, F. Lipparini, F. Egidi, J. Goings, B. Peng, A. Petrone, T. Henderson, D. Ranasinghe, V. G. Zakrzewski, J. Gao, N. Rega, G. Zheng, W. Liang, M. Hada, M. Ehara, K. Toyota, R. Fukuda, J. Hasegawa, M. Ishida, T. Nakajima, Y. Honda, O. Kitao, H. Nakai, T. Vreven, K. Throssell, J. A. Montgomery, Jr., J. E. Peralta, F. Ogliaro, M. Bearpark, J. J. Heyd, E. Brothers, K. N. Kudin, V. N. Staroverov, T. Keith, R. Kobayashi, J. Normand, K. Raghavachari, A. Rendell, J. C. Burant, S. S. Iyengar, J. Tomasi, M. Cossi, J. M. Millam, M. Klene, C. Adamo, R. Cammi, J. W. Ochterski, R. L. Martin, K. Morokuma, O. Farkas, J. B. Foresman, and D. J. Fox. *Gaussian 09, Rev. D.01* (Gaussian Inc., Wallingford, USA, CT, 2009).
7. K. Duanmu, B. W., A. V. Marenich, C. J. Cramer and D. G.Truhlar. *CM5PAC, Rev, 2015.* (University of Minnesota, Minneapolis, USA, MN, 2015).
8. Hirshfeld, F. L. Bonded-atom fragments for describing molecular charge densities. *Theor. Chem. Acc.* **44**, 129–138 (1977).
9. Duanmu, K. & Truhlar, D. G. Partial Ionic Character beyond the Pauling Paradigm: Metal Nanoparticles. *J. Phys. Chem. C* **118**, 28069–28074 (2014).
10. Kim, Y., Mohrig, J. R. & Truhlar, D. G. Free-Energy Surfaces for Liquid-Phase Reactions and Their Use To Study the Border Between Concerted and Nonconcerted α,β-Elimination Reactions of Esters and Thioesters. *J. Am. Chem. Soc.* **132**, 11071–11082 (2010).
11. Kentzinger, E., Krutyeva, M. & Rücker, U. GALAXI: Gallium anode low-angle x-ray instrument. *JLSRF.* **2**, 61 (2016).
12. Su, Y., Nemkovskiy, K. & Demirdiş, S. DNS: Diffuse scattering neutron time-of-flight spectrometer. *JLSRF.* **1**, 27 (2015).
13. Goracci, G., Arbe, A., Alegria, A., Su, Y., Gasser, U., Colmenero, J. Structure and component dynamics in binary mixtures of poly(2-(dimethylamino)ethyl methacrylate) with water and tetrahydrofuran: A diffraction, calorimetric, and dielectric spectroscopy study. *J. Chem. Phys.***144**, 154903 (2016).
